# Deep learning on routine full-breast mammograms enhances lymph node metastasis prediction in early breast cancer

**DOI:** 10.1038/s41746-025-01831-8

**Published:** 2025-07-10

**Authors:** Daqu Zhang, Looket Dihge, Pär-Ola Bendahl, Ida Arvidsson, Magnus Dustler, Julia Ellbrant, Kim Gulis, Malin Hjärtström, Mattias Ohlsson, Cornelia Rejmer, David Schmidt, Sophia Zackrisson, Patrik Edén, Lisa Rydén

**Affiliations:** 1 Centre for Environmental and Climate Science, Computational Science for Health and Environment, Lund University, Lund, Sweden; 2 Department of Clinical Sciences, Division of Surgery, Lund University, Skåne University Hospital, Lund, Sweden; 3 Department of Plastic and Reconstructive Surgery, Skåne University Hospital, Malmö, Sweden; 4 Lund University Cancer Centre, Lund University, Lund, Sweden; 5 Department of Clinical Sciences, Division of Oncology, Lund University, Lund, Sweden; 6 Centre for Mathematical Sciences, Division of Computer Vision and Machine Learning, Lund University, Lund, Sweden; 7 Department of Translational Medicine, Diagnostic Radiology, Lund University, Lund, Malmö, Sweden; 8 Department of Surgery and Gastroenterology, Skåne University Hospital, Malmö, Sweden; 9 Department of Surgery, Kristianstad Central Hospital, Kristianstad, Sweden; 10 Department of Clinical Sciences, Anesthesiology and Intensive Care, Lund University, Lund, Sweden; 11 Department of Medical Imaging and Physiology, Skåne University Hospital, Malmö, Sweden

**Keywords:** Breast cancer, Radiography, Image processing, Computational models, Machine learning

## Abstract

With the shift toward de-escalating surgery in breast cancer, prediction models incorporating imaging can reassess the need for surgical axillary staging. This study employed advancements in deep learning to comprehensively evaluate routine mammograms for preoperative lymph node metastasis prediction. Mammograms and clinicopathological data from 1265 cN0 T1–T2 breast cancer patients (primary surgery, no neoadjuvant therapy) were retrospectively collected from three Swedish institutions. Compared to models using only clinical variables, incorporating full-breast mammograms with preoperative clinical variables improved the ROC AUC from 0.690 to 0.774 (improvement: 0.001–0.154) in the independent test set. The combined model showed good calibration and, at sensitivity ≥90%, achieved a significantly better net benefit, and a sentinel lymph node biopsy reduction rate of 41.7% (13.0–62.6%). Our findings suggest that routine mammograms, particularly full-breast images, can enhance preoperative nodal status prediction. They may substitute key predictors such as pathological tumor size and multifocality, aiding patient stratification before surgery.

## Introduction

Breast cancer is the most commonly diagnosed cancer and a leading cause of cancer death worldwide^1^, with distant metastases responsible for 76% of breast cancer deaths^2^. Accurate staging of the axillary lymph node (ALN) is critical for optimizing treatment and thus for improving prognosis^3–5^ for patients diagnosed with early-stage invasive breast cancer. As the gold standard method, sentinel lymph node biopsy (SLNB) is routinely performed for patients with clinically negative (cN0) nodes. Although SLNB reduces the extent of surgery and mitigates arm morbidity compared to axillary lymph node dissection (ALND)^6,7^, it is still associated with postoperative complications, including impaired arm function^8^. Moreover, the majority of cN0 patients have low-risk tumors, node-negative disease, and excellent prognosis. For these patients, SLNB offers no therapeutic benefits but potential side effects. As a result, there is a current trend toward de-escalation of axillary surgery^9,10^, with emerging evidence indicating that SLNB can be safely omitted in certain clinically and sonographically node-negative patients^11,12^. Nevertheless, reliable noninvasive methods for accurately assessing the risk of ALN metastasis in these patients are still lacking.

Previous studies have extensively investigated the use of clinicopathological characteristics to predict axillary nodal status preoperatively in early-stage breast cancer, employing a range of machine learning methods from linear models to advanced deep learning algorithms^13–16^. Despite these efforts, discrimination performance remains deficient. For certain predictors such as tumor size, lymphovascular invasion (LVI), and multifocality, preoperative assessments are less informative and less accurate compared to the pathological assessment observed after surgery^17–19^. However, many studies rely on postoperative data, which may not reflect true predictive performance in real preoperative clinical settings. Nonetheless, these studies suggest that additional variables are needed to improve the nodal status prediction.

Ongoing research has thus investigated information-rich imaging modalities and demonstrated great potential in preoperatively evaluating axillary nodes when combining them with molecular profiles^20^. Prediction models incorporating ultrasonography (US) or magnetic resonance imaging (MRI) with clinical predictors demonstrated excellent performance^21–25^. Axillary US is the primary tool to locally stage the axilla and is currently included in the diagnostic work-up for primary breast cancer^26^. Breast MRI including the axilla, has more global views and demonstrates comparable performance to US^27^. However, US assessments are highly dependent on operator expertise, leading to variability in the results, while MRI has limited accessibility and higher costs, constraining its clinical applications.

Conversely, mammography, as the primary imaging modality recommended for all breast cancer patients, is universally implemented as part of the standard diagnostic work-up and is routinely available globally. Its widespread use, standardization, accessibility and low cost make it particularly well-suited for development of broadly applicable prediction models. While mammography is primarily used for breast imaging, it also indirectly provides valuable insights into nodal assessment by capturing key features such as surrounding tissue characteristics, tumor size, and multifocality—factors which are known to be associated with ALN metastases. Despite its central role in early breast cancer management, however, its contribution in ALN staging has received comparatively little attention and remains underexplored. Radiomic nomograms based on mammograms of region of interest (ROI) focusing on breast tumors have shown improved performance in predicting axillary metastasis in small study cohorts^28–30^, but their effectiveness still requires validation in larger populations. Although peritumoral regions add value for US-based nodal metastasis prediction^31,32^, the value of these regions on mammograms has not been confirmed, and the comprehensive information provided by full-breast mammograms remains unexplored. Additionally, ROI-based models require precise tumor detection and poses challenges in patients with multifocal breast lesions, as it can be difficult to decide which tumor should be analyzed.

Utilizing standard mammograms for ALN staging is challenging due to limited axillary visualization^33^. Moreover, analyzing high-resolution full-breast digital mammograms using traditional radiomics is ineffective, as these methods rely on manually retrieved features and have constrained scalability. Deep learning (DL) algorithms, with advantages of adaptive feature learning, scalability, and flexibility, show great potential for full-breast mammogram processing. In particular, the vision transformer^34^, employing a self-attention mechanism, is highly effective in aggregating spatial information and capturing global interactions, making it a promising approach for enhancing full-breast mammogram modeling. Additionally, transfer learning, empowered by domain-aligned self-supervised learning (SSL)^35,36^, was employed to prevent overfitting and accelerate model training.

In this study, we develop DL tools to improve full-breast mammogram modeling for predicting ALN status in patients with early breast cancer having surgery as primary treatment, alongside key predictors of nodal metastasis—tumor size, LVI status, and multifocality—which are challenging to assess preoperatively. Additionally, we assess the benefit of incorporating mammograms from early-stage breast cancer patients across multiple clinical sites to enhance the preoperative prediction of ALN metastasis. Our goal is to advance noninvasive nodal staging methods, enabling more accurate preoperative stratification and better guiding decisions regarding axillary surgery.

## Results

**Fig. 1:**
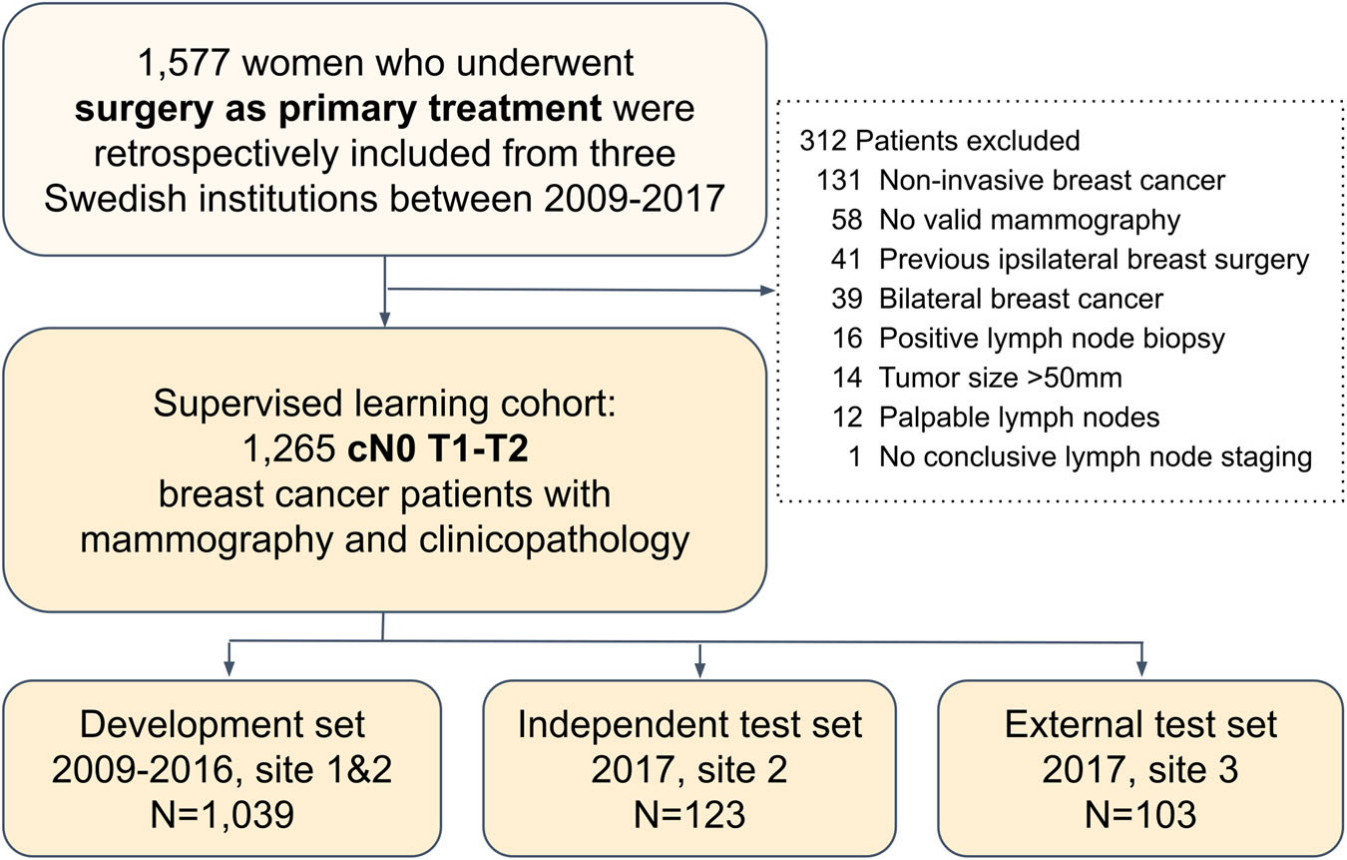
Patient selection process. The study cohort construction and patient exclusion criteria are presented. cN0 clinical node-negative, T invasive tumor grade.

Initially, 1577 women diagnosed with breast cancer were included in the study. Only patients who underwent primary surgery and axillary nodal staging were eligible for inclusion. Patients who had received neoadjuvant systemic therapy were excluded. The selection of patients for supervised learning (SL) is presented in Fig. 1. In total, 1265 patients were identified. Of those, 1039 patients diagnosed between 2009 and 2016 from sites 1 and 2 were assigned to the model development set (training and validation), whereas 123 patients diagnosed in 2017 from site 2 were allocated to the independent test set and 103 patients in 2017 from site 3 were allocated to the external test set. All model selection was based on double cross-validation (as illustrated in Supplementary Fig. 1) in the development set. End-to-end evaluation of DL and post hoc interpretation were performed in the independent test set. The external test set was revealed to lack representativeness based on the statistical analysis (Supplementary Table 1). Therefore evaluation of the external test set is not presented in the result section, instead it is provided in Supplementary Fig. 2.

We present our results by first summarizing the clinicopathological characteristics for the SL study cohort. We then present the end-to-end performance of the developed DL pipeline and demonstrate the added value of mammography in predicting lymph node metastasis (LNM). Next, we describe the detailed results of model selection for key DL components, including advanced self-supervised learning (SSL) methods and the Transformer neck module. Finally, we interpret the developed DL model through feature importance analysis and activation map visualization.

### Univariable analysis of patient characteristics

As shown in Table 1, a total of 1162 women diagnosed with primary invasive breast cancer who underwent both mammography and primary surgical treatment were included for SL development (*n* = 1039, site 1 and 2) and internal test (*n* = 123, site 2). Of these, 348 patients were diagnosed with LNM (prevalence 30%). Overall, the mean age at diagnosis was 62.8 years, the mean tumor size was 15.6 mm, and the positive rate of LVI was 17%. The majority of patients were diagnosed through mammography screening and presented with grade II invasive carcinoma of no special type (NST) and luminal A-like (LumA) subtype.

**Table 1:** Patient characteristics of the supervised learning cohort

Variables/Predictors	All (*n* = 1162)	Node negative (*n* = 814)	Node positive (*n* = 348)	*p* value	Effect size
Age, years, mean(SD)	62.8 (±11.7)	63.7 (±11.3)	60.9 (±12.5)	<0.001	−0.237^*d*^
Missing	0	0	0		
BMI, mean(SD)	26.5 (±4.8)	26.6 (±4.7)	26.3 (±5.1)	0.489	−0.047^*d*^
Missing	37	24	13		
Menopausal status, No. (%)					
Postmenopausal	910 (82)	660 (85)	250 (75)	<0.001	0.116^*V*^
Premenopausal	201 (18)	118 (15)	83 (25)		
Missing	51	36	15		
Mode of detection, No. (%)					
Symptomatic	457 (39)	277 (34)	180 (52)	<0.001	0.166^*V*^
Mammographic	705 (61)	537 (66)	168 (48)		
Missing	0	0	0		
Histological type, No. (%)					
NST	908 (78)	623 (77)	285 (82)	0.035	0.054^*V*^
ILC	150 (13)	107 (13)	43 (12)		
Others	104 (9)	84 (10)	20 (6)		
Missing	0	0	0		
Histological grade, No. (%)					
I	287 (25)	216 (27)	71 (20)	0.020	0.058^*V*^
II	551 (48)	385 (48)	166 (48)		
III	312 (27)	202 (25)	110 (32)		
Missing	12	11	1		
ER status, No. (%)					
Negative	89 (8)	72 (9)	17 (5)	0.019	0.069^*V*^
Positive	1069 (92)	738 (91)	331 (95)		
Missing	4	4	0		
PgR status, No. (%)					
Negative	179 (15)	140 (17)	39 (11)	0.009	0.077^*V*^
Positive	979 (85)	670 (83)	309 (89)		
Missing	4	4	0		
HER2 status, No. (%)					
Negative	983 (89)	695 (90)	288 (88)	0.514	0.020^*V*^
Positive	118 (11)	80 (10)	38 (12)		
Missing	61	39	22		
Ki67 status, No. (%)					
Negative	595 (53)	435 (55)	160 (48)	0.019	0.070^*V*^
Positive	525 (47)	350 (45)	175 (52)		
Missing	42	29	13		
St Gallen surrogate molecular subtype, No. (%)					
LumA	639 (59)	462 (61)	177 (55)	0.004	0.064^*V*^
LumB	261 (24)	165 (22)	96 (30)		
HER2+	118 (11)	80 (10)	38 (12)		
TNBC	67 (6)	56 (7)	11 (3)		
Missing	77	51	26		
Variables/Outcomes					
Tumor size, mm, mean(SD)	15.6 (±8.1)	14.2 (±7.6)	18.9 (±8.2)	<0.001	0.580^*d*^
Missing	0	0	0		
Multifocality of invasive foci, No. (%)					
No	886 (76)	656 (81)	230 (66)	<0.001	0.156^*V*^
Yes	276 (24)	158 (19)	118 (34)		
Missing	0	0	0		
LVI status, No. (%)					
Negative	845 (83)	651 (89)	194 (67)	<0.001	0.264^*V*^
Positive	172 (17)	78 (11)	94 (33)		
Missing	145	85	60		
No. LNMs, mean(SD)	0.7 (±1.9)	0.0 (±0.0)	2.4 (±2.8)	<0.001	1.274^*d*^
Missing	0	0	0		

Comparisons were made between patients with or without lymph node metastasis (LNM). The significance level was set at *p* = 0.05, and a nontrivial effect size was defined as Cohen’s ∣*d*∣ ≥ 0.50 for continuous variables and as Cramer’s ∣*V*∣ ≥ 0.30, ≥0.21 and ≥0.17 for categorical variables with 1, 2, and 3 degrees of freedom, respectively.

*NST* no special type, *ILC* invasive lobular carcinoma, *LumA* luminal A-like, *LumB* luminal B-like, *TNBC* triple negative breast cancer, *LVI* lymphovascular invasion, *No. LNMs* number of LNMs.

Eleven preoperative variables were identified as predictors, and five postoperative variables including LNM status were used as model outcomes. A comparison of these characteristics between patients with and without LNM is presented in Table 1. Patients with LNM were younger, more frequently premenopausal, and had a higher prevalence of symptomatically presented breast tumors. These tumors were characterized by higher histological grade, higher rates of NST and luminal B-like (LumB) subtypes, and higher positive rates of ER, PgR, and Ki67. However, none of the preoperative predictors exhibited a nontrivial effect size, with only marginal nontrivial effects for age (60.9 vs. 63.7; *p* < 0.001; *d* = −0.237) and mode of detection (52% vs. 34% symptomatic presentations; *p* < 0.001; *V* = 0.166). Regarding postoperative outcomes, patients with positive LNM exhibited a larger tumor size, a larger number of invasive foci, and a higher prevalence of LVI. Tumor size demonstrated a substantially nontrivial effect size of 0.580 (18.9 mm vs. 14.2 mm; *p* < 0.001). Additionally, marginal nontrivial effects were observed for LVI status (33% vs. 11%; *p* < 0.001; *V* = 0.264) and multifocality (34% vs. 19%; *p* < 0.001; *V* = 0.156).

A comparison between the internal independent test set and the development set is presented in Supplementary Table 2. Although significant distribution shifts of Ki67, St Gallen subtype, LVI and LNM statuses were revealed, the sets show reasonable agreement on effect sizes for nodal status. A comparison between the development and the external set (*n* = 103, site 3) is provided in Supplementary Table 1. The external test set had significantly higher BMIs, smaller tumor sizes, and lower positive rates of multifocality, LVI, and LNM. In contrast to the development set, when comparing nodal positive with nodal negative patients, LNM showed insufficient effect sizes in age, mode of detection, multifocality, and LVI status. Moreover, the effect size of the top-1 predictor, tumor size, was dramatically reduced from 0.588 in the development set to 0.361 in the external test set. This observation of weak effectiveness of both preoperative and postoperative variables is unusual, particularly when referring to existing literature^37^.

### End-to-end performance of LNM prediction

**Fig. 2:**
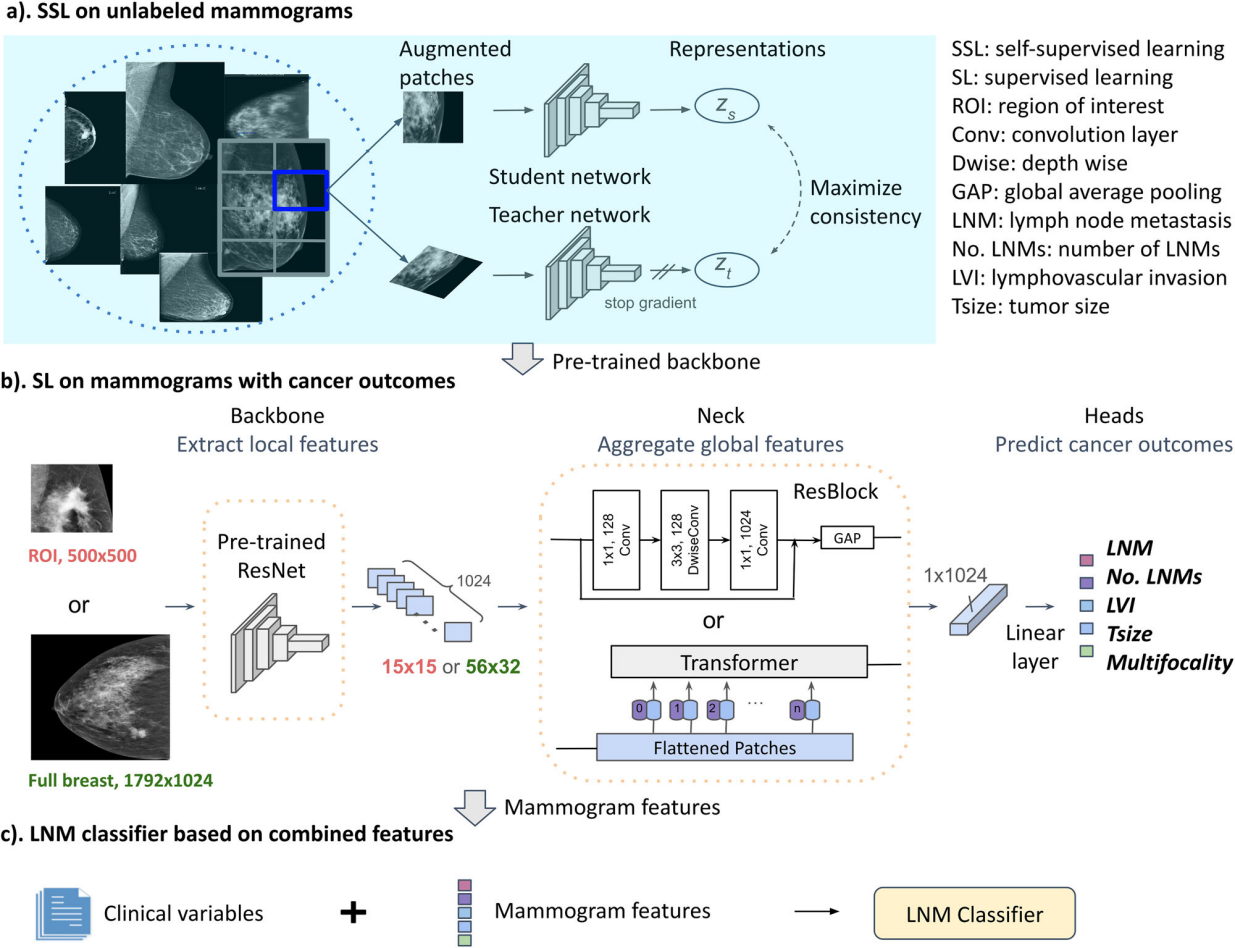
Deep learning (DL) workflow. The study employed a three-step DL approach. **a** SSL was conducted on unlabeled mammograms to pretrain the backbone feature extractor by maximizing the consistency between augmentations of the same patch. Contiguous patches were tiled from high-resolution mammograms to enable the extraction of subtle variations in local breast tissue. **b** SL was performed on mammograms of all views with five cancer outcomes to establish a neck module—a global feature extractor, on top of the backbone. For the neck module, a residual block and a vision Transformer were separately evaluated on the ROI or full-breast mammograms. Tumor ROIs were annotated by an automatic detecting tool. The five cancer outcomes investigated included LNM, number of LNMs, LVI, Tsize, and multifocality. **c** The extracted mammogram features and 11 preoperative clinical variables were concatenated to collaboratively train the final LNM classifier. Mammogram preprocessing details are provided in Supplementary Section 4.

As illustrated in Fig. 2, DL was conducted in three steps. (1) SSL on unlabeled mammogram patches was performed to pretrain a backbone to extract basic representations (local features). (2) Higher-level representations (global features) were further developed by a neck module through SL to predict postoperative outcomes. (3) The combination of mammography and clinicopathology features was used to develop the final classifier for predicting LNM.

**Fig. 3:**
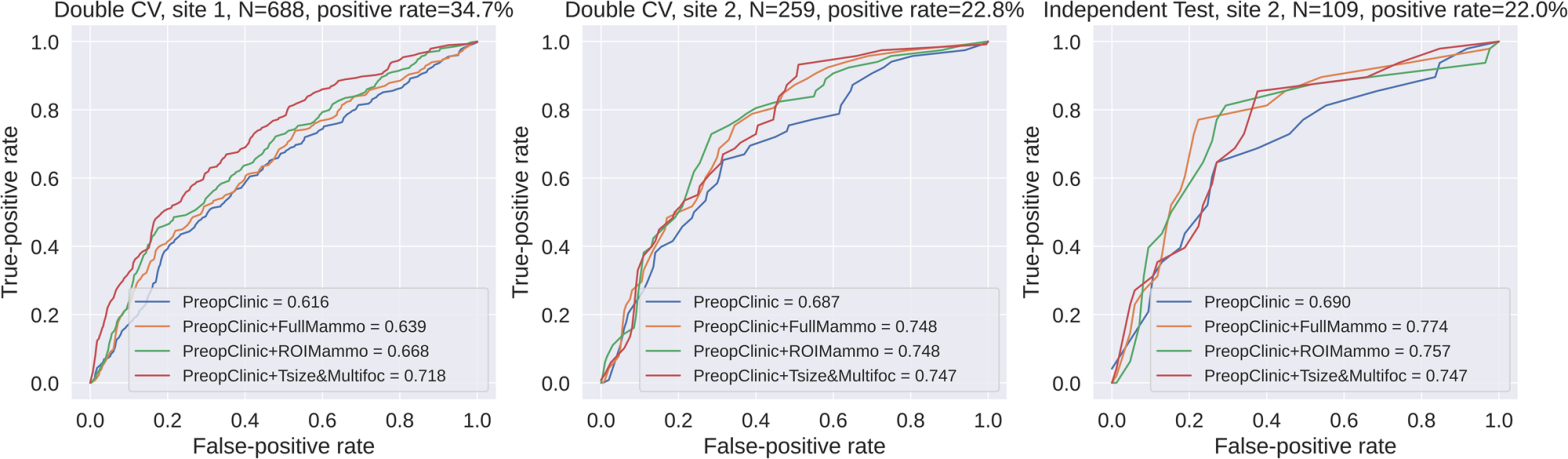
Comparisons of ROC curves for LNM prediction between models using various modalities. ROC curves of the development set (left column for site 1 and middle column for site 2) and the independent test set (right column, site 2) are presented. The number of patients, *N*, and positive rate for each set are presented in the subtitles. The areas under the curves are presented in the figure legends. LNM lymph node metastasis, PreopClinic preoperative clinicopathology, fullMammo full-breast mammogram, ROIMammo mammogram of region of interest, Tsize tumor size, Multifoc multifocality, CV cross-validation, ROC receiver operating characteristic.

The final LNM classifiers combining preoperative clinical predictors and DL mammogram features of the full breast (*PreopClinic* *+* *FullMammo*) or ROI (*PreopClinic* *+* *ROIMammo*) were compared to the classifier using only the clinical predictors (*PreopClinic*). Additionally, the two most important postoperative indicators, tumor size and multifocality, were added to the preoperative clinical modeling (*PreopClinic* *+* *Tsize&Multifoc*) to benchmark the added value of mammography in predicting LNM. DL models based on *FullMammo* and *ROIMammo* employed the same ResNet backbone (pretrained using BarlowTwins) and Transformer-neck neural network architecture. Receiver operating characteristic (ROC) curves and bootstrap the area under curve (AUC) of these models were calculated on different sets to showcase the site-dependent performance (Fig. 3 and Table 2). All bootstrap 95% confidence intervals (CIs) for model improvement and *P* values from permutation significance tests were calculated in comparison to *PreopClinic* (Table 2).

**Table 2:** Mammograms enhanced the preoperative prediction of lymph node metastasis across all datasets

Set	Model modality	ROC AUC ± SD	Improvement CI (95%)	*p* value
Development set (site 1, *N* = 688, Double CV)	PreopClinic	0.616 ± 0.023	–	–
	PreopClinic + FullMammo	0.639 ± 0.023	−0.006–0.050	0.119
	PreopClinic + ROIMammo	0.668 ± 0.022	0.018–0.083	0.002
	PreopClinic + Tsize&Multifoc	0.718 ± 0.021	0.063–0.140	<0.001
Development set (site 2, *N* = 259, Double CV)	PreopClinic	0.687 ± 0.039	–	–
	PreopClinic + FullMammo	0.748 ± 0.035	0.006–0.108	0.021
	PreopClinic + ROIMammo	0.748 ± 0.035	−0.002–0.119	0.071
	PreopClinic + Tsize&Multifoc	0.747 ± 0.032	−0.005–0.119	0.077
Independent test (site 2, *N* = 109)	PreopClinic	0.690 ± 0.063	–	–
	PreopClinic + FullMammo	0.774 ± 0.057	0.001–0.154	0.037
	PreopClinic + ROIMammo	0.757 ± 0.060	−0.021–0.143	0.142
	PreopClinic + Tsize&Multifoc	0.747 ± 0.054	−0.058–0.159	0.300

The mean and standard deviation (SD) of the receiver operating characteristic (ROC) area under the curve (AUC), as well as the confidence interval (CI) for the improvement, were calculated using bootstrap with 1000 samples. All CIs for model improvement and permutation test *p* values were calculated in comparisons to *PreopClinic*.

*CV* cross-validation, *N* number of patients, *PreopClinic* preoperative clinicopathology, *fullMammo* full-breast mammogram, *ROIMammo* mammogram of region of interest, *Tsize* tumor size, *Multifoc* multifocality.

Double cross-validation on development set 1 (site 1) showed that *ROIMammo* improved the ROC AUC from 0.616 to 0.668 (*p* = 0.002), while *FullMammo* yielded a marginal improvement (AUC of 0.639, *p* = 0.119). Neither outperformed *PreopClinic* *+* *Tsize&Multifoc* (AUC of 0.718). In development set 2 (site 2), both *FullMammo* and *ROIMammo* improved the preoperative AUCs from 0.687 to 0.748 (*p* = 0.021 and *p* = 0.071, respectively), and were comparable with *PreopClinic* *+* *Tsize&Multifoc* (AUC of 0.747). In the independent test set (site 2), *FullMammo* achieved large improvement in AUC from 0.690 to 0.774 (*p* = 0.037), and *ROIMammo* showed a marginal improvement (AUC of 0.757, *p* = 0.142). Although both models outperformed *PreopClinic* *+* *Tsize&Multifoc* (AUC of 0.747), the differences were not statistically significant. Considerable variability in model performance was observed between sites, with AUCs ranging from 0.616 to 0.718 at site 1 and from 0.687 to 0.774 at site 2. The overall result demonstrated that full-breast mammograms can achieve comparable improvement as tumor ROI patches, and the added value of mammography showed dependence on sites, with a larger improvement of *FullMammo* for site 2 and a larger improvement of *ROIMammo* for site 1.

Importantly, under the constraint that the predicting sensitivity is no less than 90%, reflecting the generally acceptable sensitivity of SLNB^38^, *PreopClinc* *+* *FullMammo* boosted the performance metrics over *PreopClinc* (improvement CI: −4.1–28.5%, *p* = 0.136 for SLNB reduction rate; improvement CI: 2.3–11.7%, *p* < 0.001 for net benefit), and demonstrated comparable performance to *PreopClinic* *+* *Tsize&Multifoc* (Table 3). Although only the improvement in net benefit reached statistical significance in the independent test set, likely due to considerable variations stemming from the small sample size and class imbalance, it is notable that models that incorporate mammogram features (both FullMammo and PreopClinc + FullMammo) consistently outperformed PreopClinc, on average, across all performance metrics. This highlights their potential for more accurate predictions. The mean and standard deviations were calculated across 1000 bootstrap samples.

**Table 3:** Mammograms largely enhanced the performance metrics for the prediction of lymph node metastasis in the independent test set at a sensitivity ≥90%

Metrics	PreopClinic	FullMammo	PreopClinic + FullMammo	PreopClinic + Tsize&Multifoc
ROC AUC	0.706 (±0.055)	**0.782** (±0.047)	**0.782** (±0.050)	0.740 (±0.051)
PR AUC	0.468 (±0.073)	**0.522** (±0.077)	0.504 (±0.077)	0.461 (±0.075)
Sensitivity (recall), %	91.0 (±2.6)	91.0 (±2.7)	91.2 (±2.9)	91.1 (±2.7)
Specificity (TNR), %	31.9 (±13.5)	**50.9** (±12.3)	50.3 (±16.4)	45.7 (±15.7)
PPV (precision), %	28.9 (±4.5)	36.4 (±6.5)	**36.8** (±8.6)	34.4 (±7.0)
NPV, %	91.5 (±3.9)	**94.9** (±1.7)	**94.9** (±2.2)	94.2 (±2.4)
Accuracy, %	45.4 (±10.2)	**60.0** (±9.3)	59.6 (±12.4)	56.0 (±11.9)
SLNB reduction rate, %	27.0 (±11.0)	41.1 (±10.2)	**41.7** (±13.0)	37.3 (±12.1)
Net benefit, %	3.6 (±1.7)	**12.8** (±2.0)	10.9 (±2.2)	9.0 (±1.8)

Performance metrics were calculated using the same independent test set as previous (site 2, *N* = 123, LNM positive rate = 22.8%), with the addition of 14 patients for whom ROI annotations were unavailable in Fig. 3 and Table 2. Mean and standard deviation were calculated across 1000 bootstrap samples. The best mean value among all models is denoted in bold. Net benefit measures the trade-off between benefit (true positives) and harm (false positives) at the threshold when sensitivity is no less than 90%. Decision curve analysis of net benefit against [0,1] threshold is provided in Supplementary Fig. 4.

*PreopClinic* preoperative clinicopathology, *fullMammo* full-breast mammogram, *Tsize* tumor size, *Multifoc* multifocality, *ROC* receiver operating characteristics, *AUC* area under the curve, *PR* precision recall, *TNR* true negative rate, *PPV* positive predictive value, *NPV* negative predictive value, *SLNB* sentinel lymph node biopsy, SLNB reduction rate = (True Negatives + False Negatives)/All.

### Self-supervised learning improved the representations for predicting cancer outcomes

**Fig. 4:**
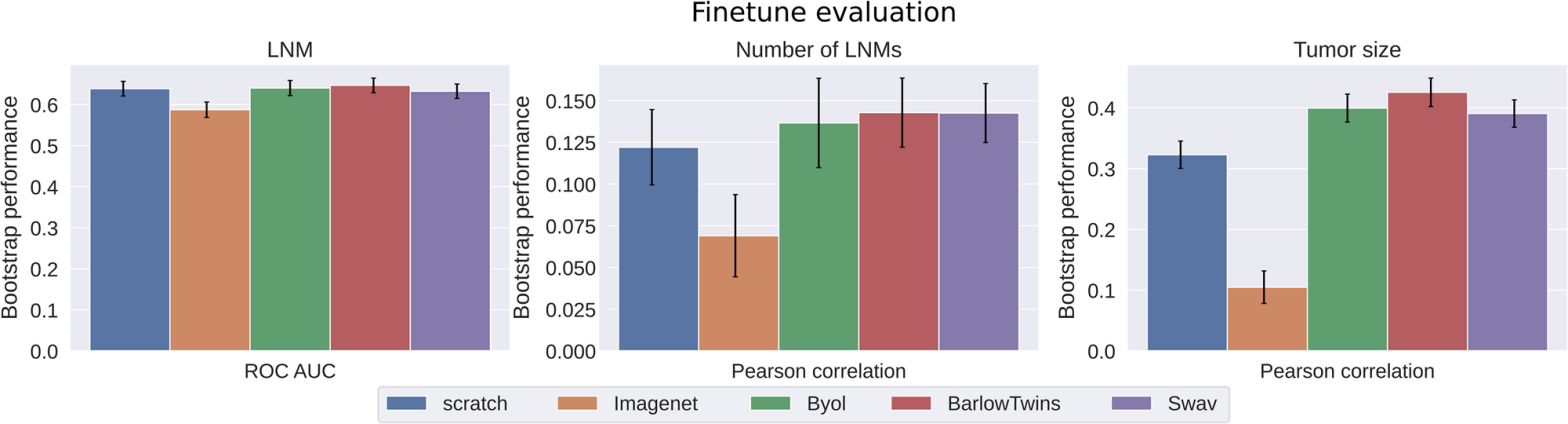
Evaluation of three state-of-the-art self-supervised learning (SSL) methods. SSL on unlabeled mammograms enhanced the representations for predicting cancer outcomes. The mean and standard deviation (error bars) were calculated across 1000 bootstrap samples. LNM lymph node metastasis, ROC AUC area under the receiver operating characteristics curve.

To determine the effectiveness of domain-adapted SSL in learning mammogram representation, three advanced SSL methods were compared to random initialization and benchmarked by ImageNet pretraining through finetune evaluation protocols (Supplementary Section 2) on cancer-related postoperative outcomes. As shown in Fig. 4, bootstrap performances revealed that domain-adapted SSL had substantially higher transfer learning ability than the ImageNet pretraining and outperformed the random initialization for all tasks.

### Transformer showed superior performance in aggregating global mammogram features

**Fig. 5:**
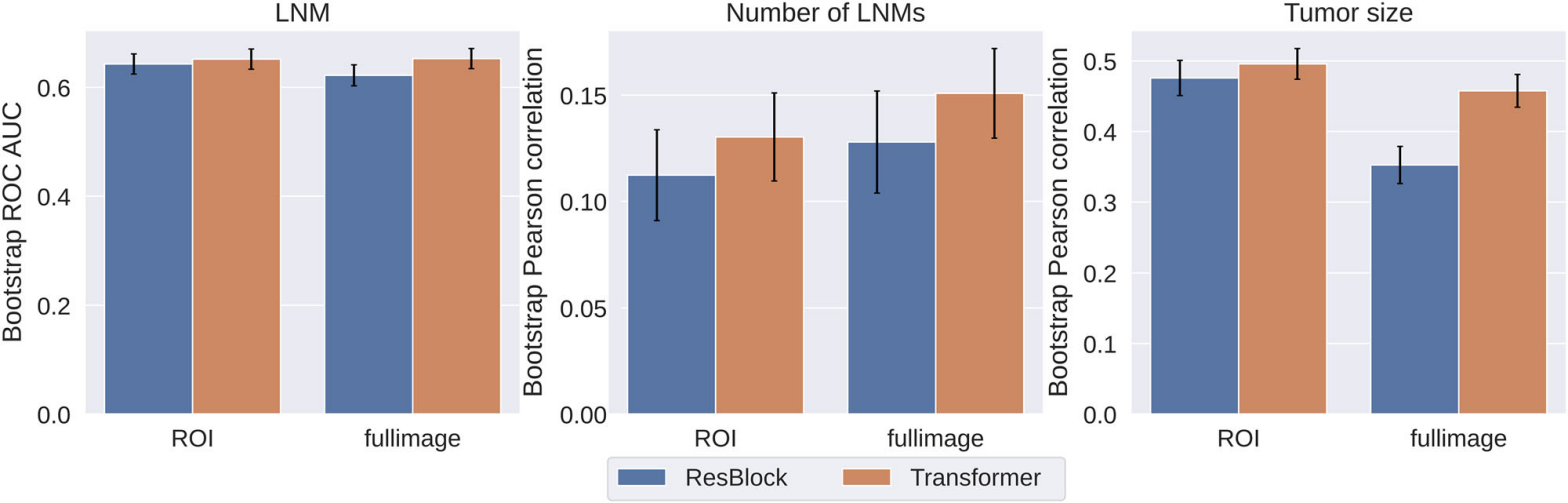
Comparisons of Transformer and ResBlock for mammogram modeling. Transformer outperformed ResBlock in full-breast and ROI-based mammogram modeling for predicting cancer outcomes. The mean and standard deviation (error bars) were calculated across 1000 bootstrap samples. ROI region of interest, LNM lymph node metastasis, ROC receiver operating characteristics, AUC area under the curve.

To evaluate the ability of modeling global correlations of mammograms, especially for full breast, the performances of postoperative outcomes were compared between Transformer and ResBlock models at different scopes of full-breast or ROI (Fig. 5). The results showed that the Transformer model outperformed ResBlock for both full-breast and ROI-based mammogram modeling for all tasks, in particular for predicting number of LNMs and pathological tumor size, and especially using full-breast image modeling. The evaluation results for the prediction of LVI and multifocality are presented in Supplementary Fig. 5 for SSL methods and Supplementary Fig. 6 for global mammogram feature extraction approaches.

### Model interpretation analyses

**Fig. 6:**
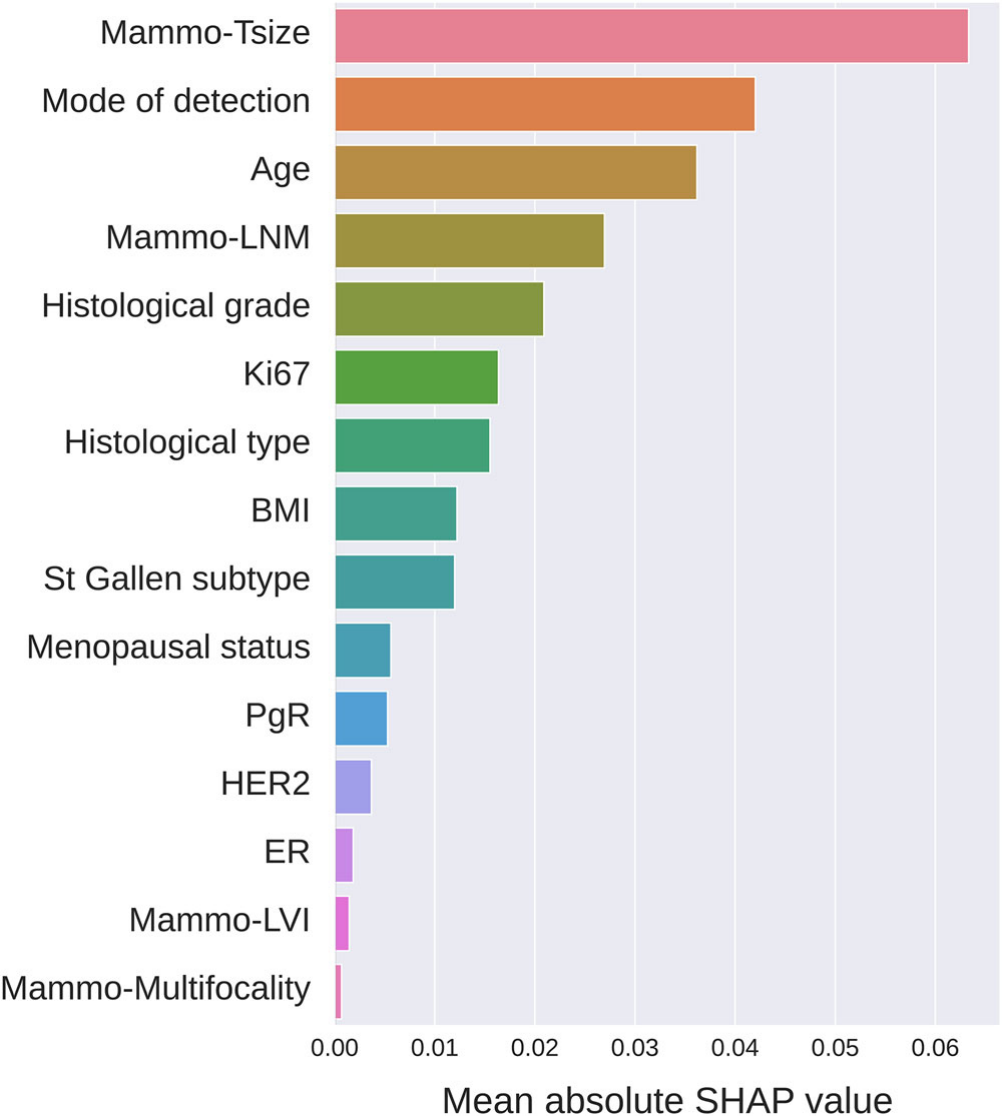
Feature importance analysis. Mammogram features played an important role in predicting lymph node metastasis. Feature importance estimated by the mean absolute SHAP value of the combined model of preoperative predictors and full-breast mammogram features is presented.

Figure 6 demonstrates the feature importance estimated by the SHAP value for the combined model of preoperative clinicopathology and full-breast mammogram features in the independent test set. Preoperative predictors and mammographic features were ranked by the average importance across all 25 double cross-validation Transformer models, with decreasing importance from top to bottom. Mammography tumor size by Transformer models (denoted as Mammo-Tsize) ranked at the top and exhibited a significant lead over the rest of the predictors. The mode of detection and age were the second and third most important predictors, respectively, which is consistent with the univariable effect size analysis shown in Table 1. Mammo-LNM prediction (combined binary and continuous prediction of LNM) was the fourth of the most important predictor. This highlights the important role of mammography in preoperative prediction of LNM in early breast cancer.

**Fig. 7:**
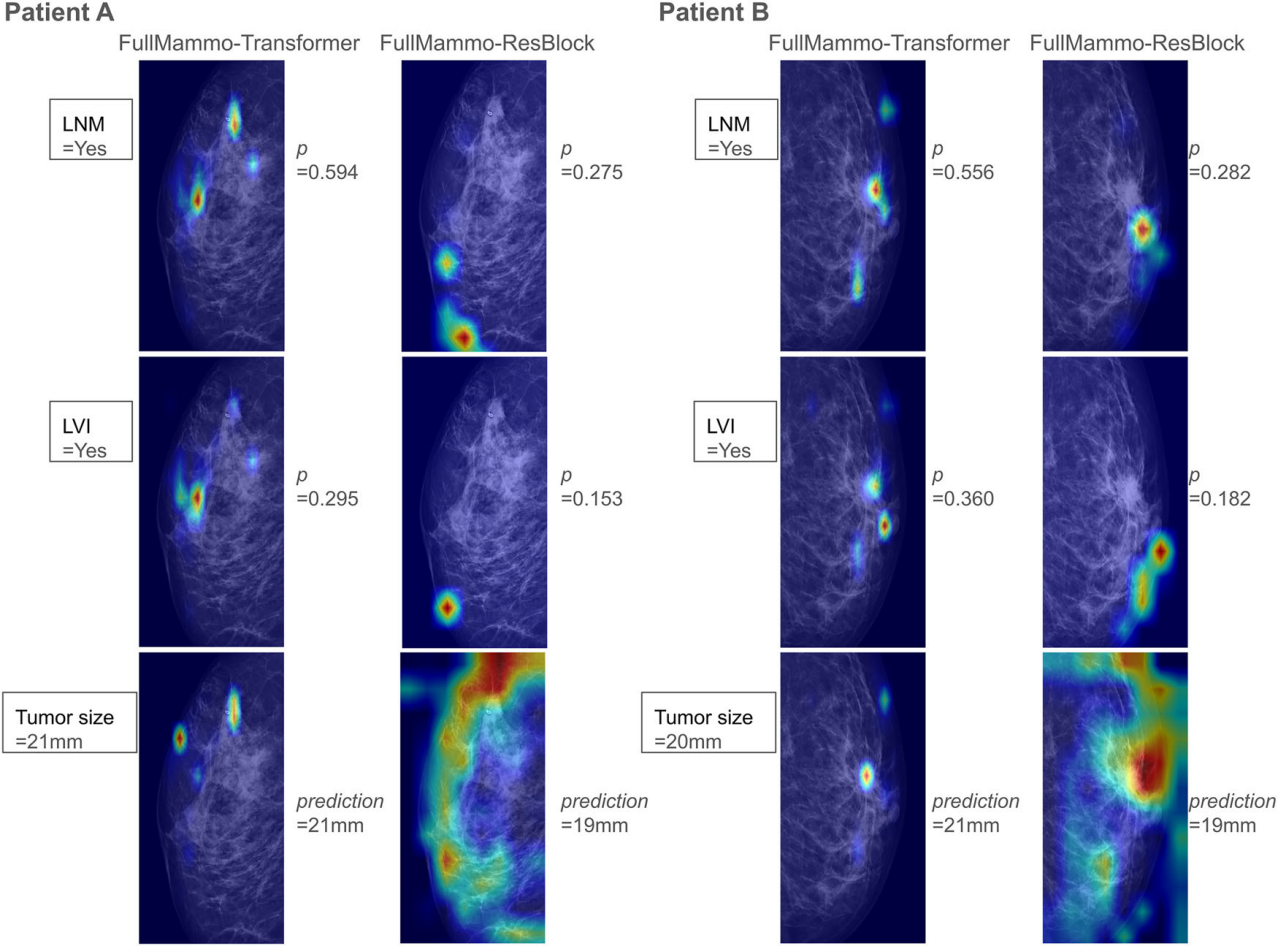
Visualization of activation maps. Grad-CAM was used to interpret the mammogram patterns. The Transformer localized tumors more accurately for tumor size prediction and assigned higher importance to tumor and peritumor regions for predicting lymph node metastasis (LNM) and lymphovascular invasion (LVI) compared to the ResBlock model. Both selected patients **A** and **B** were LNM positive and LVI positive showing mediolateral views with visible lesions to demonstrate the activation maps of the Transformer and the ResBlock model (columns 1–2 for patient **A** and columns 3–4 for patient **B**). Model predictions were denoted as *p*. The expectations of LNM and LVI predictions for the Transformer model were 0.239 and 0.158, respectively, and 0.240 and 0.178 for the ResBlock model.

To further understand the mammogram patterns in DL prediction for LNM, LVI, and tumor size (Tsize), qualitative analysis using Grad-CAM was conducted (Fig. 7). Multifocality prediction was excluded due to poor performance and number of LNMs was excluded due to overlap with LNM prediction. Mediolateral views of full-breast mammogram from two LNM- and LVI-positive patients with visible breast lesions were selected to demonstrate the activation maps for the Transformer and ResBlock models. The activation maps of Tsize prediction showed more accurate tumor localization by the Transformer model compared to ResBlock. When predicting LNM and LVI status, the Transformer assigned higher importance to tumor and peri-tumor regions, whereas the ResBlock model lost focus on tumors. This partially explains the superior performance of the Transformer and highlights its ability to capture global imaging patterns relevant to LVI and LNM prediction.

## Discussion

In this study, we developed and validated DL models based on preoperatively available clinicopathological predictors and full-breast digital mammogram features to predict LNM status and comprehensively evaluated the added value of routine mammograms to preoperatively predict LNM in multicenter cohorts of cN0 T1–T2 breast cancer patients having primary surgery. The results demonstrate that advances in DL techniques, including domain-adaptive pretraining through SSL and sophisticated spatial modeling empowered by a Transformer, significantly improve imaging analysis, particularly for full-breast mammograms. Our findings reveal that routine mammograms can substantially enhance the preoperative diagnosis of LNM in early-stage breast cancer, and are as informative as key postoperative pathological indicators, such as tumor size and multifocality. Notably, full-breast mammograms exhibited overall comparable predictive performance to tumor region-focused models and showed favorable discriminating ability in the independent test set, underscoring its clinical applicability. These results highlight the great value of routine mammograms in staging LNM before surgery and aiding in preoperative patient stratification for axillary management by increasing the SLNB reduction rate from 27% to 42%.

To the best of our knowledge, this is the first multicenter study for LNM status prediction in cN0 T1-T2 breast cancer patients undergoing surgery as primary treatment using advanced DL techniques on full-breast digital mammograms. Yang et al.^28^, Tan et al.^29^, and Haraguchi et al.^30^ have reported the added value of mammogram tumor segmentations in predicting LNM, using radiomics-based models in relatively small and single-center study cohorts (ranging from 77 to 216 patients), and the effectiveness was not externally validated. These studies had a wide range of ROC AUCs from 0.608 to 0.891. Direct comparisons with these studies were infeasible due to large differences in study populations regarding age at diagnosis, tumor grade and size, and prevalence of nodal metastasis. Compared to our clinical models, incorporating full-breast mammograms improved the preoperative prediction of LNM across all sites (Table 2 and Supplementary Fig. 2). This approach achieved ROC AUCs ranging from 0.673–0.879 (bootstrap 95% CI), exhibited good calibration, and demonstrated greater net benefits and higher SLNB reduction rates at sensitivity ≥90% in the independent test set (Supplementary Figs. 3 and 4).

While many imaging techniques other than axillary US (which however has high dependency on operators^39^ and shows large variance in discrimination^26^) are considered inappropriate for axillary staging^40^, breast lesion imaging using these techniques have been investigated for improved diagnosis of nodal metastasis. Adjunctive modalities of color Doppler US identifying irregular blood flow and shear wave elastography depicting tissue stiffness have been combined with conventional US^22^ for ALN prediction. The DL models based on multiple US modalities largely improved the preoperative ROC AUC from 0.630–0.825 to 0.843–0.961. However, breast lesions deeper than 3 cm in depth or larger than 3.5 cm in diameter were excluded due to the attenuation and limited width of the US probe. Advanced MRI techniques^24,25,41^, have been investigated and demonstrated satisfying ROC AUCs of 0.708–0.883 for predicting LNM status. Such imaging techniques require extra costs and are not always available, and MRI is added to the diagnostic workflow much later in the process than mammography. One similarity shared across these studies is that tumor ROIs need to be manually outlined by radiologists before extracting radiomics or DL features. Thus, our proposed full-breast modeling approach could be applied to other imaging modalities for further enhancement.

Compared to our ROI-based models, full-breast mammogram models exhibited higher performance for predicting LNM and LVI status but lower performance for predicting multifocality and tumor size (Fig. 5 and Supplementary Fig. 6). This suggests that LNM and LVI may be associated with global imaging patterns which were overlooked by ROI-based models focusing only on tumors. Supporting this finding, Grad-CAM visualization demonstrated that Transformer accurately localized tumor areas for predicting tumor size and assigned high importance to both tumor and peri-tumor regions for LNM and LVI identification. Using global information, the full-breast mammogram models could eliminate the need for tumor ROIs by automatic detection tools (such as Transpara^42^) or potentially reduce the reliance on radiologists for annotations, and also resolve the challenges of selecting which ROI should be analyzed for patients with multifocal breast lesions. Consequently, our full-breast mammogram approach has the potential to enhance efficiency and broaden the applicability of the proposed tool for predicting nodal status preoperatively in the absence of ROI annotations.

The innovative design of the Transformer neck, leveraging the attention mechanism, enhanced global feature extraction by emphasizing important features in high-resolution, full-breast mammograms and significantly improved the predicting performance compared to ResBlock (Fig. 5). It is worth noting that efficient training techniques for Transformer were indispensable when modeling full-breast mammograms with only a few thousand samples. Shifted patch tokenization and locality self-attention^43^ reduced the need for a large dataset by enabling the model to learn meaningful attention patterns more efficiently. Compared to ImageNet pretraining, domain-adapted pretraining largely improved the effectiveness in representation learning and transfer learning for downstream tasks (Fig. 4 and Supplementary Fig. 5). Additional cancer-free mammograms did not improve the representation learning for cancer-related downstream tasks, as shown in Supplementary Table 3. This suggested that such a distribution shift introduced by cancer-free patterns might cancel the benefit of enhanced diversity.

SHAP value-based feature importance analysis revealed that, among all preoperative clinical variables and mammogram features, tumor size predicted by the mammogram model was the most significant contributor to LNM prediction. The mode of detection, i.e., mammography or symptomatic presentation, ranked as the second most important variable, also encoding important mammographic information. Mammography LNM prediction ranked fourth following age at diagnosis. The high SHAP values of mammogram features underscores the crucial role mammography plays in preoperative LNM prediction.

Our study also investigated the additional value of mammography for predicting LVI, multifocality, and tumor size. The full-breast mammogram model exhibited a ROC AUC of 0.662 in predicting LVI, with a sensitivity of 89.4% and a specificity of 28.5% (Supplementary Table 4). Comparing to the preoperative clinical model, mammograms did not improve the predicting metrics for LVI identification, indicating the high predictive ability of clinicopathological data. The authors in ref. ^44^ reported similar performance of mammography for predicting LVI when applying expert morphology criteria, with a high specificity of 98.8% but a deficient sensitivity at 14.3%. As for predicting multifocality, the overall performance is poor. Importantly, full-breast mammogram modeling via the Transformer achieved a high accuracy in predicting tumor size, with Pearson correlations of 0.458 in the development set and 0.620 in the independent test set (Supplementary Table 5). In comparison, previous research using tomosynthesis (3D mammography) reported Pearson correlations of 0.57 (95% CI: 0.36–0.72)^45^ and 0.41 with both digital mammograms and tomosynthesis^46^.

The multicentric evaluation revealed significant site- and time-dependent variability in the added value of mammography as well as the overall prediction ability of clinical variables. Among sites 1–3, site 2 demonstrated the best LNM prediction for PreopClinic and PreopClinic + Tsize&Multifoc models with ROC AUCs of 0.687 and 0.747, respectively (Fig. 3). In contrast, site 3 showed the poorest performance with ROC AUCs of 0.584 for PreopClinic and 0.633 for PreopClinic + Tsize&Multifoc (Supplementary Fig. 2). This observation aligns with the univariable analysis, where predictors of site 2 exhibited the largest overall effect sizes on LNM status (Supplementary Table 2), whereas the smallest effect sizes were observed in site 3 (Supplementary Table 1). Regarding the predictive ability of mammography, sites 1 and 3 yielded the same performance with a ROC AUC of 0.614 and performed much worse than site 2 (ROC AUCs of 0.700 for the development set and 0.776 for the independent test set). This study collected patient data over a period from 2009–2017, during which we observed a decline in LNM prevalence over time (from 34.7% at site 1, 2009–2012, to 22.0–22.8% at site 2, 2010–2017, Fig. 3). This decline most likely reflects temporal shifts in diagnostic work-up, including earlier detection through public mammography screening. The increasingly imbalanced distribution of LNMs may have contributed to variability in model performance through complex interactions with other site- and time-dependent factors. Further research is needed to investigate the potential factors behind this observed site-dependence including calendar years for diagnosis, mammography equipment, and advancements of clinical tests.

De-escalation strategies for axillary surgery have evolved dramatically in the last decades by abandoning ALND as an axillary staging method in favor of SLNB. The landmark Z0011 trial^6^ demonstrated that ALND could be safely omitted in selected patients with 1–2 positive sentinel nodes undergoing breast-conserving surgery and radiation. More recently, the large multicenter SENOMAC trial^47^ extended the generalizability of this approach to include patients undergoing mastectomy. The SENOMAC trial demonstrated that omission of completion ALND in patients with clinically node-negative T1–T3 breast cancer with one or two sentinel-node macrometastases did not compromise recurrence-free survival. Accurate noninvasive prediction of lymph node metastasis is essential for optimizing axillary staging and management in breast cancer patients. Reflecting that surgical axillary staging has no or limited effect on locoregional recurrence and breast-cancer-specific mortality^48,49^ in defined subgroups of patients with early nodal disease burden and cN0 invasive breast cancer, the American Society of Clinical Oncology guidelines^50^ have recommended that SLNB can be omitted for these patients but endorsed case-by-case decision-making. The SOUND^12^ trial demonstrated the noninferiority of abstaining SLNB compared to performing SLNB in terms of 5-year distant disease-free survival in patients with primary breast cancer with T1 tumors and a normal ultrasound after breast-conserving surgery. It has to be proven if this finding is true also for patients with larger tumors and if long-term follow-up will change the conclusions from the trial. Importantly, randomized clinical trials, such as the already published INSEMA^11^ and BOOG 2013-08^51^ trials, are further assessing the oncologic safety of omitting SLNB in cN0 breast cancer patients with larger tumors and the final results are soon to be presented. This shift toward de-escalation of surgical staging underscores the need for accurate noninvasive tools to identify patients at low risk for nodal metastasis to allow a case-by-case-evaluation. Remarkably, our DL models incorporating mammograms suggested a SLNB reduction rate of 41.7% in the independent test set enabling omission of surgeries for low-risk patients. The proportion of patients in which SLNB can be omitted following the application of our DL model is in the range of the CE-IVD marked Merlin^*TM*^ test for melanoma, which is currently being evaluated in a prospective registered trial^52^. DL, with its ability to integrate imaging, clinicopathological and genomic data^53^, holds great promise for improving preoperative prediction of LNM and tailoring treatment to individual patients.

This study has several limitations which need to be addressed. Surprisingly, the external test set was not representative in terms of important clinical predictors for nodal status like tumor size, which reduced its usability. Further evaluation on independent external sets need to be conducted to validate the findings. This large site-dependent variability in LNM prediction was an important finding but the underlying factors are unclear. Although the Transformer neck module outperformed traditional convolutional neural networks in aggregating global mammographic features, a fully end-to-end Transformer solution for mammograms was disregarded in the present study due to the extremely high computational cost and large demand of patients’ data for training. Although the previous studies^42,54^ on Transpara reported tumor detection accuracy comparable to that of radiologists, the ROI annotations used in this study were not guaranteed to be completely correct in detecting tumors. In this study, some preoperative predictors (histological grade, histopathological type, and molecular profile) were assessed during the final pathological examination. Although these variables can be accurately estimated using core needle biopsy, further studies need to be conducted to evaluate the predictive ability using core needle biopsy measurements in a real clinical setting.

Our work demonstrates that leveraging advancements of DL on full-breast digital mammograms combined with clinicopathological characteristics significantly enhanced the preoperative prediction of axillary lymph node metastasis and may compensate for the most important postoperative predictors such as tumor size and multifocality. Therefore, our method provides a noninvasive and efficient way for preoperative risk stratification of lymph node metastasis in clinically node negative breast cancer patients, potentially identifying >40% of them as candidates for abstention of SLNB. The multicentric evaluation revealed significant variability in predicting nodal status, highlighting the need for further research to identify potential underlying factors contributing to these site-dependent effects.

## Methods

### Study cohort

Initially, 1577 women diagnosed with breast cancer, who underwent surgery as primary treatment but did not receive neoadjuvant therapy, were included in the study and their clinicopathological tabular data were retrieved. The inclusion criteria were histologically confirmed invasive breast cancer; primary surgery treatment without prior neoadjuvant therapy; valid axillary staging; and availability of preoperative full-field digital mammography acquired within 200 days before surgery. The patient selection for the final study cohort is presented in Fig. 1. The exclusion criteria were as follows: noninvasive breast cancer (ductal carcinoma in situ, lobular carcinoma in situ, and other in situ cancers), prior ipsilateral breast surgery, bilateral breast cancer, biopsy-confirmed ALN positivity, tumor size >50 mm, palpable lymph nodes, incongruent or missing ALN staging, and absence of valid preoperative mammograms (defined as images corresponding to the correct breast side and obtained within the specified preoperative window). In total, 1265 patients were identified and divided into development and test sets according to the site and year of diagnosis.

The research and data usage agreements were reviewed and approved by the Ethical Committee at Lund University (LU 2013/340, 2022-00878-02). The study was conducted using data sources from the Lund University Hospital, Malmö University Hospital and Kristianstad Hospital, and was approved by the internal review board of Region Skåne (KVB). The use of the MBTST-DM dataset^55^ was conducted in accordance with the approvals and amendments outlined in Dnr 2009/770 and Dnr 2017/326. The need for informed consent was waived by the Ethical Committee at Lund University and The Ethics Review Board for this study in accordance with the legislation. Construction and reporting of the prediction models followed the guidelines of Transparent Reporting of a Multivariable Prediction Model for Individual Prognosis or Diagnosis (TRIPOD) + AI^56^.

### Mammography examination

Full-field digital mammograms were retrospectively retrieved from hospital databases and four open-source datasets (as presented in Table 4). Individual sets 1–3 were collected from three institutions in Sweden, i.e., Lund University Hospital from 2009–2012 (site 1), Malmö University Hospital from 2010–2017 (site 2), and Kristianstad Hospital in 2017 (site 3). Sets 1–2 were used for supervised learning (SL), development and testing, as well as self-supervised learning (SSL), and set 3 was used only for SL testing. The open-source sets 4–7 (CBIS-DDSM^57^, MIAS^58^, INbreast^59^, MBTST-DM^55^) provided only mammograms and were included to enhance diversity for SSL and thus improve representation learning on unlabeled mammograms. CBIS-DDSM, MIAS, and INBreast consist of major cancer and minor normal cases. Additionally, more than 14k cancer-free women enrolled for the MBTST-DM, a breast cancer screening trial embedded in the screening program from 2010–2015^60^, were used to investigate the added value of cancer-free mammograms to representation learning for cancer-related downstream tasks.

**Table 4:** Dataset summary

Set	Usage	Source	Private	Year	#Subjects	Subject categories	Clinico-pathology	Number of images	Manufacturer	View
1	SSL,SL	Lund	Yes	2009–2012	778	Cancer	Yes	4790	99% GE	CC, MLO ML
2	SSL, SL	Malmö	Yes	2010–2017	750	Cancer	Yes	10,783	99% Siemens	CC, MLO ML
3	Test	Kristianstad	Yes	2017	103	Cancer	Yes	473	100% GE	CC, MLO ML
4	SSL	CBIS-DDSM^a^	No	1999	1566	Cancer, normal	No	3032	Unknown	CC, MLO
5	SSL	MIAS^b^	No	2003	161	Cancer, normal	No	322	Unknown	MLO
6	SSL	INBreast^c^	No	2011	115	Cancer, normal	No	410	Unknown	CC, MLO
7	SSL	MBTST-DM^d^	No	2010–2015	14,669	Normal	No	59,365	100% Siemens	CC, MLO

Seven datasets from three acquired and four open sources were collected for this study. Supervised learning (SL) was developed and tested on sets 1–2, where both mammograms and clinical predictors and outcomes were available. Set 3 was the external test set to evaluate SL. Sets 4–7, with large number of unlabeled mammograms, were only used for self-supervised learning (SSL). In sets 4–6, cancer patients comprised the majority. For sets 1–2, the numbers of subjects and images refer to those used for SSL, while detailed patient selection for the SL cohort is presented in Fig. 1.

*GE* general electric, *CC* craniocaudal, *ML* mediolateral, *MLO* mediolateral oblique.

^a^Curated Breast Imaging Subset of Digital Database of Screening Mammography^57^.

^b^Mammographic Imaging Analysis Society database^58^.

^c^InBreast database^59^.

^d^Malmö Breast Tomosynthesis Screening Trial—Digital Mammography database^55^.

The mix of cases includes both women diagnosed through population-based mammography screening and women diagnosed outside of the screening program after presenting with symptoms. Depending on the mode of diagnosis and availability, either screening mammograms, additional mammograms acquired at recall from screening, or clinical mammograms acquired after clinical referral of symptomatic women were included in the study cohort. Details of the mammography examination including vendor and view information are provided in Table 4. Mammograms of all available views (craniocaudal, mediolateral and mediolateral oblique) were used for SSL and SL. Both ROI-based and full-breast imaging models take a single image as model input and generate prediction at image level, while patient level mammogram features was calculated as the maximal prediction across multiple views.

ROI was defined as abnormal breast tissue including soft tissue lesions and calcification clusters. ScreenPoint Transpara (version 1.7), a clinically used AI-based mammographic cancer detection software, was applied to each mammogram to automatically annotate the ROI center (X and Y coordinates). Transpara reviews mammograms for signs of suspicious breast cancer. From previous publications^42,54,61^, it is known to provide detection performance comparable to that of breast radiologists. In a previous study^62^, we evaluated the accuracy of Transpara-generated annotations by comparing them with radiologist-confirmed tumor regions on the same mammography dataset (Set 1 in Table 4), and observed a concordance rate of 96.1%. Of the 1039 patients in the SL development set, 947 returned qualified ROI annotations by Transpara. ROI patches were then segmented according to the center and a large predefined ROI size of 500 × 500 pixels to ensure that all tumor tissue would be included.

### Clinicopathology data collection

Clinicopathology data, including preoperative predictors for modeling and postoperative outcomes for supervised training, were obtained from individual patient’s files following a case report form^14,63^. The preoperative clinicopathological (PreopClinic) inputs were selected based on previous studies^16,64^ and only if they can be easily and accurately assessed before surgery on core needle biopsies. These PreopClinic variables included patient characteristics and tumor characteristics, including age, BMI, menopausal status, mode of detection (mammography screening or symptomatic presentation), histological grade, histopathological type (no special type—NST, invasive lobular carcinoma—ILC, and other types of invasive carcinoma), and molecular profile (estrogen receptor—ER, progesterone receptor—PgR, HER2, Ki67, and the compiled St. Gallen surrogate molecular subtype). The outcomes that can only be accurately collected postoperatively included lymph node metastasis (LNM) status and number of LNMs, lymphovascular invasion (LVI) status, pathological tumor size, and pathological multifocality (two or more tumor foci separated by benign tissue). LNM, as the primary outcome of interest, was defined as the presence of ≥0.2 mm metastasis in the sentinel lymph node, including micro- and macrometastasis.

In this study, all histopathological variables (LVI, tumor size, multifocality, histological grade, histopathological type, and molecular profile) were assessed during the final pathological examination of the primary breast tumor and evaluated according to the Swedish Society of Pathology criteria^65^. Lymph nodes were evaluated after SLNB and/or ALND. The expressions of ER, PgR, and Ki67 were assessed by immunohistochemistry (IHC) and were dichotomized into low or high expression (ER ≥1%, PgR ≥1%, and Ki67 ≥20%). To evaluate HER2 status, IHC and in situ hybridization (ISH) were performed, and tumors were classified as HER2-positive if they had an IHC score of 3+ and/or a positive ISH test. The St. Gallen surrogate molecular subtypes included luminal A-like (LumA), luminal B-like (LumB), HER2-positive (HER2+), and triple-negative breast cancer (TNBC) according to^66^.

### Deep learning workflow

The three-step DL workflow was illustrated in Fig. 2. The DL models were developed and validated using PyTorch (1.10.1) installed under Python (v3.8.18). Detailed data preprocessing for mammography and clinicopathology data is presented in Supplementary Section 4.

SSL on unlabeled mammograms was performed to pretrain a backbone to extract basic mammogram features. Patches tiled from full-breast mammograms were randomly augmented into pairs, and the paired augmented patches were forwarded to a student and a teacher ResNet, respectively, which were then optimized by maximizing the consistency between the student and teacher representations (Fig. 2a). According to recent benchmark studies of SSL on diverse medical image datasets^35,36,67^, three state-of-the-art SSL methods, BYOL^68^, BarlowTwins^69^, and SwAV^70^, were investigated. These algorithms share similarities in that they maximize the consistency between different augmentations of the same image. BYOL adopts the exponential moving average of the student network as the teacher network and minimizes the mean squared error. BarlowTwins uses identical student and teacher networks and optimizes the identity cross-correlation matrix. SwAV implements a cross-entropy loss and applies multicrop augmentation strategies. The implementation details of SSL are provided in Supplementary Section 2.

SL to predict postoperative cancer outcomes was further developed to extract global mammogram features. On top of the ResNet backbone, the neck module of residual blocks (ResBlock) or a vision Transformer was separately developed to aggregate the tumor ROI or full-image features to extract useful task-specific representations (Fig. 2b). For the ResBlock neck, one block was designed for the ROI and two blocks for full-breast images, to allow for a sufficient reception field before the global average pooling operator. Narrowed middle-layer (reduced from 1024 to 128 channels) and depthwise convolution were adopted to greatly reduce the number of parameters to prevent overfitting. For the Transformer neck, ROI and full-breast mammogram features were flattened in their spatial dimensions with each feature vector (1 × 1024) functioning as a patch embedding. Shifted patch tokenization and locality self-attention proposed in ref. ^43^ were applied to the vision Transformer to improve performance on a small dataset. The global representations in a 1D vector of 1024 elements were then fed to five predicting heads. The neck module was shared across five tasks and was trained simultaneously, predicting LNM (binary classification and regression of the continuous number of positive nodes), LVI status (binary classification), tumor size (regression), and multifocality (binary classification). These five postoperative outcomes were designed to allow for extracting relevant features for metastasis diagnosing while learning more diverse aspects of mammograms to prevent overfitting compared to single-task learning. The implementation details of SL are provided in Supplementary Section 3.

Finally, mammogram features were concatenated to the preoperative clinicopathological predictors to collaboratively model a classifier predicting LNM. To balance the size of mammogram features with the 11 clinical variables, the five cancer outcome predictions were used, extracted after completing the training of the neck. To obtain patient-level mammogram features, an ensemble was performed by calculating the average prediction of double-cross-validation models and then the maximal prediction across multiple views from the same patient. A simple MLP with one hidden layer of 128 neurons was implemented and trained using default hyperparameters (batch size = 32, dropout = 0.2, optimizer = AdamW, learning rate = 0.01, and weight decay = 0.0001).

### Model evaluation and post hoc analyses

Double cross-validation, as illustrated in Supplementary Fig. 1, was adopted to minimize data uncertainty of small sample sizes by allowing for validation in the entire development set, while reducing overfitting by inserting an inner validation loop. Double cross-validation on the development set was used for model selection of mammogram analysis, with inner-loop validation for early stop and outer-loop validation for tuning the hyperparameters and optimizing the DL framework. Post hoc analyses including feature importance analysis, activation map visualization, calibration, and decision curves analyses, were conducted in the independent test set. Calibration curves were generated using the Scikit-learn calibration package.

Receiver operating characteristic (ROC) and precision-recall (PR) AUCs were used to evaluate the overall performance of binary classification tasks, including the prediction of LNM status, LVI status, and multifocality, while Pearson correlation was used for evaluation of regression tasks, including the prediction of tumor size and number of LNMs. To estimate the clinical utility of the developed models for LNM prediction, the specificity, positive predictive value (PPV) or precision, negative predictive value (NPV), accuracy, net benefitsuggested, and SLNB reduction rate, i.e., (True Negatives + False Negatives)/All, were reported at thresholds optimized for a sensitivity of no less than 90%. Decision curve analysis of the trade-off between benefits (true positive) and harms (false positive) was performed using the net benefit measure defined in ref. ^71^. Mean and standard deviations of all performance metrics were reported across 1000 bootstrap samples.

The SHAP explainer^72^ was used to estimate feature importance for the LNM classifier based on clinical variables and learned mammographic features. Feature importance was calculated as the mean absolute value of raw SHAP values across individual samples.

GradCam^73^ was applied to visualize activation maps for the developed DL models. Activation maps are coarse localization maps highlighting the important regions in the image for predicting the target. The last convolutional layer before the global average pooling operator was used to visualize the ResBlock models while the last convolutional layer before the last attention block was used for the Transformer models.

### Statistical analysis

In the univariable analysis of patient characteristics, Student’s *t* test with unequal variance was utilized to detect statistical differences between continuous variables, and the *χ*^2^ test was used for categorical variables. Student’s *t* and *χ*^2^ tests were two-tailed, and the significance level was set at *p* = 0.05. Furthermore, an effect size analysis was conducted to indicate the magnitude of the detected differences. For continuous variables, the effect size was evaluated by the difference in means relative to the standard deviation, denoted as Cohen’s *d*^74^. A nontrivial effect size for continuous variables was defined as ∣*d*∣ ≥ 0.50^75^. The effect size for categorical variables was evaluated using Cramer’s *V*. A nontrivial effect size for categorical variables was defined as ∣*V*∣ ≥ 0.30, ∣*V*∣ ≥ 0.21, and ∣*V*∣ ≥ 0.17 for 1, 2, and 3 degrees of freedom, respectively (degrees of freedom were calculated by (*r* − 1)*(*c* − 1), where *r* and *c* are the numbers of rows and columns of the contingency table)^76^.

The two-tailed permutation test^77^ was used to detect statistical differences in performance metrics between two models, and the significance level was set at *p* = 0.05. The bootstrap CI (95%) of performance differences between two models was calculated using the reversed percentile interval^78^.

## Supplementary information

Supplementary Information

## Data Availability

The open source mammography datasets used in this study are available at: CBIS-DDSM database, https://wiki.cancerimagingarchive.net/pages/viewpage.action?pageId=22516629; MIAS database, https://www.repository.cam.ac.uk/items/b6a97f0c-3b9b-40ad-8f18-3d121eef1459; INbreast database, https://www.kaggle.com/datasets/martholi/inbreast; MBTST-DM, https://datahub.aida.scilifelab.se/10.23698/aida/mbtst-dm. The private mammography and clinicopathology data used in this study cannot be deposited in a public repository due to ethical prohibitions but are available from the lead contact upon reasonable request.

## Code availability

All codes used for modeling are available at https://github.com/dorazhang93/Mammography-nils/tree/main/projects/Mammography.
