# Supplementary material for: Deep learning on routine full-breast mammograms enhances lymph node metastasis prediction in early breast cancer
Source: NPJ Digit Med. 2025 Jul 10;8:425. doi: 10.1038/s41746-025-01831-8 (PMC12246406; doi:10.1038/s41746-025-01831-8)
Supplement: Supplementary file 1 — Supplementary Information [file 41746_2025_1831_MOESM1_ESM.pdf]

Deep Learning on Routine Full-Breast Mammograms Enhances Lymph Node Metastasis Prediction in Early Breast Cancer

Supplementary materials

Supplementary Section 1: Image augmentation details

For **self-supervised learning** (SSL), we followed the original augmentation schemes proposed specifically by each SSL algorithm [1–3]. The transformations were extended as follows:

- Random Resized Crop.
- Random horizontal flip (p=0.5).
- Auto-adjusts image contrast.
- Adjust image brightness with a relative magnitude range of -0.5 to 0.5.
- Adjust image sharpness with a relative magnitude range of -0.5 to 0.5.
- Equalize the image histogram (p=0.5).

For **supervised learning**, we applied the following augmentation scheme:

- Random Crop: The region of interest (ROI) patches were cropped with a random size (0.5 to 1.0) of the original size and a random aspect ratio (3/4 to 4/3) of the original aspect ratio. Full-breast images were cropped with a random size (0.5 to 0.9) of the original size and a random aspect ratio (1/2 to 3/4) of the original aspect ratio.
- Resize: ROIs were resized to 500 x 500 pixels, and the full-breast images were resized to 1,792 x 1,024 pixels.
- Random horizontal flip (p=0.5).
- Auto-adjusts image contrast.
- Rotate images with an angle range of -20 to 20 degrees.
- Adjust image brightness with a relative magnitude range of -0.25 to 0.25.
- Adjust image sharpness with a relative magnitude range of -0.25 to 0.25.
- Equalize the image histogram (p=0.5).

Supplementary Section 2: Implementation details of self-supervised learning (SSL)

To avoid overfitting, a ResNet-50 with reduced width scaling (channel sizes reduced by half) was designed as the backbone feature extractor for mammograms. Hybrid transfer learning using lightweight models was proved to be more efficient than the

standard ImageNet architectures [4]. Accordingly, only the first block of ResNet backbone reused the ImageNet pretrained weights and the remaining slimmed blocks were randomly initialized and pretrainned using SSL. Three state-of-the-art SSL methods, BYOL [1], BarlowTwins [2] and SwAV [3] were implemented using the OpenMMLab library [5]. All these SSL models were trained for 80 epochs with batch size 512 and distributed over 2 A40 48GB GPUs. The LARS optimizer [6] with base learning rate 0.6, momentum 0.9 and weight decay 1e-6 was adopted. A linear warmup learning rate schedule was applied for the first 5 epochs and a cosine annealing schedule was applied subsequently. Each method was originally proposed with its specific augmentation schemes and we followed the original workflow but used mammography-adjusted transformations described in Supplementary Section 1.

Downstream tasks, i.e., predicting cancer outcomes, were used to assess the effectiveness of SSL algorithms, using the well-established finetune evalutation protocols. In a fintune setting, the pretrained backbone was first frozen and multi-task classifiers (neck and head modules) were developed on top and parameters were optimized. Next, the backbone was unfrozen and the entire model were trained end-to-end. This measures the transfer learning ability of the pretrained model.

Supplementary Section 3: Implementation details of supervised learning (SL)

SL of five cancer outcomes were conducted on ROIs or full-breast images using the deep learning (DL) pipeline consisting of backbone, neck module and predicting heads. Training included two stages: 1. freeze the pretrained backbone and optimize the neck module and predicting heads; 2. unfreeze the backbone and finetune the DL model end-to-end. Both SL steps adopted the same image augmentation scheme described in Supplementary Section 1. SL were implemented using the OpenMMLab library [5].

- SL stage 1: Both the Transformer and ResBlock models were trained for 30 epochs with batch size 72 on single GPU. The AdamW optimizer [7] with base learning rate 0.001, momentum factors of 0.9 and 0.999, and weight decay 0.001 was adopted. A step learning rate schedule with decay by 0.95 every epoch was applied.
- SL stage 2: Both the Transformer and ResBlock models were trained for 10 epochs with batch size 72 on single GPU. The AdamW optimizer [7] with base learning rate 0.0001, momentum factors of 0.9 and 0.999, and weight decay 0.001 was adopted. A step learning rate schedule with decay by 0.95 every epoch was applied.

Supplementary Section 4: Data preprocessing details

Mammography

The image selection process for SSL included removing mammograms that failed to be re-coded or were presented with implantation. Only full-field digital mammography was included. For SL on cancer related tasks, mammography images without tumors, or taken after or more than 200 days before the operation date were excluded. Preprocessing procedures applied to both SSL and SL included recoding raw mammograms to a black background with fixed resolution at 0.094 pixel/mm, projection to 0-255 grayscale using sigmoid transformation (using DICOM intensity window), cropping out artifacts and background using a bounding box calculated based on pixel intensity.

For SSL, full-breast mammograms were tiled into high-resolution patches. This patch-based input allows for extraction of small variations in local breast tissue, while reduced computation per image enables large training batch sizes — a key factor in the success of SSL methods. Patch learning can also prevent a network from taking

advantage of the overall shapes of the breast and by passing the pathological patterns. Patches of two resolutions of 640 x 640 and 480 x 480 pixels were extracted. Any patches that contained more than 45% backgrounds or had low variation (standard deviation of pixel values $\leq$15, with pixel value ranges form 0-255 grascale) were removed. As for SL, both full-breast mammograms and ROI patches detected using a clinically used AI-based software were investigated.

Image augmentation is essential for enhancing DL performance on images, which is even more important for medical applications to prevent overfitting due to limited data size. Thus, the standard pipeline of cropping, flipping and rotation was extended by a collection of effective mammography-specific augmentations namely shifts of contrast, brightness and sharpness, respectively, as well as histogram equalization [8]. Implementation details are provided in Supplementary Section 1.

Clinicopathology

Numerical clinical inputs including age and BMI were normalized using the standard scaler provided by the scikit-learn library, while the remaining categorical clinical inputs were one-hot labeled, meaning each category was transformed into a binary vector, where only one element is '1' (representing the presence of the category) and all other elements are '0' . Missing variables were imputed using the mean value for the numerical and the mode for the categorical. To prevent information leakage from the test set, normalization and missing-value imputation were conducted using the statistics of the development set only.

Supplementary Table 1: The external test set was not representative

	Variables	Development set All (n=1,039)	Development set Positive (n=320)	Development set Negative (n=719)	P value	Effect size	External test set All (n=103)	External test set Positive (n=20)	External test set Negative (n=83)	P value	Effect size	P value	Effect size
Predictors	**Age**, y, mean(SD)	63.0 (±11.7)	61.4 (±12.3)	63.7 (±11.4)	0.006	-0.190	62.2 (±10.4)	61.0 (±8.9)	62.4 (±10.8)	0.559	-0.132	0.458	0.069
	**BMI**, mean(SD)	26.4 (±4.7)	26.4 (±5.1)	26.4 (±4.6)	0.886	-0.010	27.7 (±5.4)	28.1 (±5.1)	27.6 (±5.4)	0.697	0.095	0.019	-0.276
	**Menopausal status**, No. (%)												
	Postmenopausal	811 (82)	233 (76)	578 (84)	0.002	0.098			-				
	Premenopausal	183 (18)	74 (24)	109 (16)					-				
	**Mode of detection**, No. (%)												
	Symptomatic	411 (40)	163 (51)	248 (34)	< 0.001	0.155	36 (35)	6 (30)	30 (36)	0.605	0.051	0.361	0.027
	Mammographic	628 (60)	157 (49)	471 (66)			67 (65)	14 (70)	53 (64)				
	**Histological type**, No. (%)												
	NST	817 (79)	260 (81)	557 (77)	0.089	0.048	88 (85)	19 (95)	69 (83)	0.333	0.103	0.180	0.039
	ILC	134 (13)	42 (13)	92 (13)			7 (7)	0 (0)	7 (8)				
	Others	88 (8)	18 (6)	70 (10)			8 (8)	1 (5)	7 (8)				
	**Histological grade**, No. (%)												
	I	261 (25)	64 (20)	197 (28)	0.008	0.069	29 (28)	4 (20)	25 (30)	0.538	0.078	0.556	0.023
	II	488 (47)	152 (48)	336 (47)			51 (50)	10 (50)	41 (49)				
	III	279 (27)	103 (32)	176 (25)			23 (22)	6 (30)	17 (20)				
	**ER status**, No. (%)												
	Negative	84 (8)	16 (5)	68 (10)	0.014	0.076	10 (10)	1 (5)	9 (11)	0.428	0.078	0.575	0.017
	Positive	951 (92)	304 (95)	647 (90)			93 (90)	19 (95)	74 (89)				
	**PgR status**, No. (%)												
	Negative	164 (16)	37 (12)	127 (18)	0.012	0.078	18 (17)	4 (20)	14 (17)	0.741	0.033	0.667	0.013
	Positive	871 (84)	283 (88)	588 (82)			85 (83)	16 (80)	69 (83)				
	**HER2 status**, No. (%)												
	Negative	867 (89)	260 (87)	607 (89)	0.360	0.029	95 (92)	18 (90)	77 (93)	0.678	0.041	0.269	0.034
	Positive	111 (11)	38 (13)	73 (11)			8 (8)	2 (10)	6 (7)				
	**Ki67 status**, No. (%)												
	Negative	557 (56)	155 (50)	402 (58)	0.023	0.072	67 (65)	14 (70)	53 (64)	0.605	0.051	0.073	0.054
	Positive	440 (44)	152 (50)	288 (42)			36 (35)	6 (30)	30 (36)				
	**St Gallen surrogate molecular subtype**, No. (%)												
	LumA	581 (60)	167 (57)	414 (62)	0.004	0.068	71 (69)	13 (65)	58 (70)	0.311	0.108	0.247	0.036
	LumB	208 (22)	79 (27)	129 (19)			16 (16)	5 (25)	11 (13)				
	HER2+	111 (12)	38 (13)	73 (11)			8 (8)	2 (10)	6 (7)				
	TNBC	63 (7)	10 (3)	53 (8)			8 (8)	0 (0)	8 (10)				
Outcomes	**Tumor size**, mm, mean(SD)	15.7 (±8.2)	19.1 (±8.3)	14.2 (±7.7)	< 0.001	0.588	14.1 (±6.6)	16.0 (±7.5)	13.6 (±6.3)	0.201	0.361	0.022	0.196
	**Multifocality of invasive foci**, No. (%)												
	No	792 (76)	210 (66)	582 (81)	< 0.001	0.166	90 (87)	17 (85)	73 (88)	0.721	0.035	0.010	0.076
	Yes	247 (24)	110 (34)	137 (19)			13 (13)	3 (15)	10 (12)				
	**LVI status**, No. (%)												
	Negative	756 (84)	179 (69)	577 (91)	< 0.001	0.276	98 (97)	18 (95)	80 (98)	0.514	0.065	0.001	0.108
	Positive	139 (16)	81 (31)	58 (9)			3 (3)	1 (5)	2 (2)				
	**No. LNMs**, mean(SD)	0.8 (±2.0)	2.5 (±2.9)	0.0 (±0.0)	< 0.001	1.263	0.3 (±0.7)	1.6 (±0.7)	0.0 (±0.0)	< 0.001	2.259	< 0.001	0.226
	**LNM status**, No. (%)												
	Negative	719 (69)	0 (0)	719 (100)	< 0.001	1.000	83 (81)	0 (0)	83 (100)	< 0.001	1.000	0.016	0.071
	Positive	320 (31)	320 (100)	0 (0)			20 (19)	20 (100)	0 (0)				

Comparisons were made between node positive *vs.* node negative patients within the development set and the external test set respectively. Additionally, the overall differences between the development and the external test set were compared. P values and effect sizes were calculated. The significance level was set at $P = 0.05$, and a nontrivial effect size for continuous variables was defined as Cohen's $|d| \geq 0.50$, and for categorical variables was defined as Cramer's $|V| \geq 0.30$, ≥ 0.21 and ≥ 0.17 for 1, 2, and 3 degrees of freedom, respectively. The patients from the external test set had significantly higher BMIs (27.7 *vs.* 26.4; $P = 0.019$; $d = -0.276$), smaller tumor sizes (14.1 *vs.* 15.7; $P = 0.022$; $d = 0.196$), and lower positive rates of multifocality (12% *vs.* 23%; $P = 0.010$; $V = -0.076$), LVI (2% *vs.* 15%; $P = 0.001$; $V = 0.108$) and LNM status (19% *vs.* 30%; $P = 0.016$; $V = 0.071$). Contradictory to the development set, comparing node-positive patients to the node-negative, LNM had insufficient effect sizes on age, mode of detection, multifocality or LVI status. Moreover, the effect size of the top-1 predictor, tumor size, was dramatically reduced from 0.588 in the development set to 0.361 in the external test set.

Supplementary Table 2: Clinical characteristics showed strong effect sizes on lymph node metastasis status in the independent test set

	Variables	Development set: All (n=1,039)	Development set: Positive (n=320)	Development set: Negative (n=719)	P value	Effect size	Independent test set: All (n=123)	Independent test set: Positive (n=28)	Independent test set: Negative (n=95)	P value	Effect size	P value	Effect size
Predictors	**Age**, y, mean(SD)	63.0 (±11.7)	61.4 (±12.3)	63.7 (±11.4)	0.006	-0.190	61.6 (±11.8)	54.6 (±12.5)	63.7 (±10.8)	0.001	-0.776	0.237	0.114
	**BMI**, mean(SD)	26.4 (±4.7)	26.4 (±5.1)	26.4 (±4.6)	0.886	-0.010	27.0 (±5.3)	25.7 (±5.1)	27.4 (±5.3)	0.125	-0.328	0.260	-0.119
	**Menopausal status**, No. (%)				0.002	0.098				0.002	0.285	0.421	0.024
	Postmenopausal	811 (82)	233 (76)	578 (84)			99 (85)	17 (65)	82 (90)				
	Premenopausal	183 (18)	74 (24)	109 (16)			18 (15)	9 (35)	9 (10)				
	**Mode of detection**, No. (%)				< 0.001	0.155				0.004	0.262	0.643	0.014
	Symptomatic	411 (40)	163 (51)	248 (34)			46 (37)	17 (61)	29 (31)				
	Mammographic	628 (60)	157 (49)	471 (66)			77 (63)	11 (39)	66 (69)				
	**Histological type**, No. (%)				0.089	0.048				0.101	0.137	0.242	0.035
	NST	817 (79)	260 (81)	557 (77)			91 (74)	25 (89)	66 (69)				
	ILC	134 (13)	42 (13)	92 (13)			16 (13)	1 (4)	15 (16)				
	Others	88 (8)	18 (6)	70 (10)			16 (13)	2 (7)	14 (15)				
	**Histological grade**, No. (%)				0.008	0.069				0.858	0.035	0.571	0.022
	I	261 (25)	64 (20)	197 (28)			26 (21)	7 (25)	19 (20)				
	II	488 (48)	152 (48)	336 (47)			63 (52)	14 (50)	49 (52)				
	III	279 (27)	103 (32)	176 (25)			33 (27)	7 (25)	26 (28)				
	**ER status**, No. (%)				0.014	0.076				0.880	0.014	0.111	0.047
	Negative	84 (8)	16 (5)	68 (10)			5 (4)	1 (4)	4 (4)				
	Positive	951 (92)	304 (95)	647 (90)			118 (96)	27 (96)	91 (96)				
	**PgR status**, No. (%)				0.012	0.078				0.353	0.084	0.290	0.031
	Negative	164 (16)	37 (12)	127 (18)			15 (12)	2 (7)	13 (14)				
	Positive	871 (84)	283 (88)	588 (82)			108 (88)	26 (93)	82 (86)				
	**HER2 status**, No. (%)				0.360	0.029				0.139	0.133	0.056	0.058
	Negative	867 (89)	260 (87)	607 (89)			116 (94)	28 (100)	88 (93)				
	Positive	111 (11)	38 (13)	73 (11)			7 (6)	0 (0)	7 (7)				
	**Ki67 status**, No. (%)				0.023	0.072				0.089	0.153	< 0.001	0.156
	Negative	557 (56)	155 (50)	402 (58)			38 (31)	5 (18)	33 (35)				
	Positive	440 (44)	152 (50)	288 (42)			85 (69)	23 (82)	62 (65)				
	**St Gallen surrogate molecular subtype**, No. (%)				0.004	0.068				0.129	0.124	< 0.001	0.096
	LumA	581 (60)	167 (57)	414 (62)			58 (48)	10 (36)	48 (51)				
	LumB	208 (22)	79 (27)	129 (19)			53 (43)	17 (61)	36 (38)				
	HER2+	111 (11)	38 (13)	73 (11)			7 (6)	0 (0)	7 (7)				
	TNBC	63 (7)	10 (3)	53 (8)			4 (3)	1 (4)	3 (3)				
Outcomes	**Tumor size**, mm, mean(SD)	15.7 (±8.2)	19.1 (±8.3)	14.2 (±7.7)	< 0.001	0.588	14.7 (±6.5)	16.9 (±6.1)	14.1 (±6.5)	0.042	0.429	0.126	0.120
	**Multifocality of invasive foci**, No. (%)				< 0.001	0.166				0.479	0.064	0.962	0.001
	No	792 (76)	210 (66)	582 (81)			94 (76)	20 (71)	74 (78)				
	Yes	247 (24)	110 (34)	137 (19)			29 (24)	8 (29)	21 (22)				
	**LVI status**, No. (%)				< 0.001	0.276				0.009	0.238	0.001	0.100
	Negative	756 (84)	179 (69)	577 (91)			89 (73)	15 (54)	74 (79)				
	Positive	139 (16)	81 (31)	58 (9)			33 (27)	13 (46)	20 (21)				
	**No. LNMs**, mean(SD)	0.8 (±2.0)	2.5 (±2.9)	0.0 (±0.0)	< 0.001	1.263	0.4 (±1.1)	1.6 (±1.7)	0.0 (±0.0)	< 0.001	1.529	0.001	0.199
	**LNM status**, No. (%)				< 0.001	1.000				< 0.001	1.000	0.066	0.054
	Negative	719 (69)	0 (0)	719 (100)			95 (77)	0 (0)	95 (100)				
	Positive	320 (31)	320 (100)	0 (0)			28 (23)	28 (100)	0 (0)				

Comparisons were made between positive *vs.* negative patients within the development set and the independent test set respectively. Additionally, the overall differences between the development and the independent test set were compared. P values and effect sizes were calculated. The significance level was set at $P = 0.05$, and a nontrivial effect size for continuous variables was defined as Cohen's $|d| \geq 0.50$, and for categorical variables was defined as Cramer's $|V| \geq 0.30$, ≥ 0.21 and ≥ 0.17 for 1, 2, and 3 degrees of freedom, respectively.

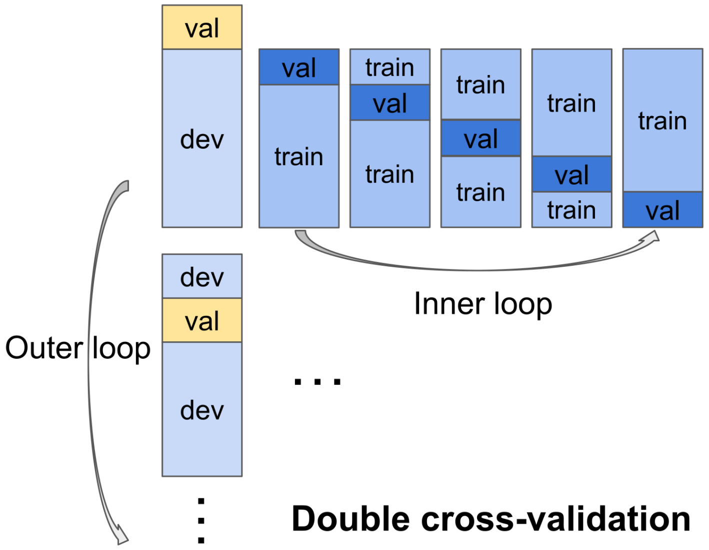

Supplementary Figure 1: Double cross-validation diagram. Both inner and outer loops were 5 fold. dev: development; val, validation.

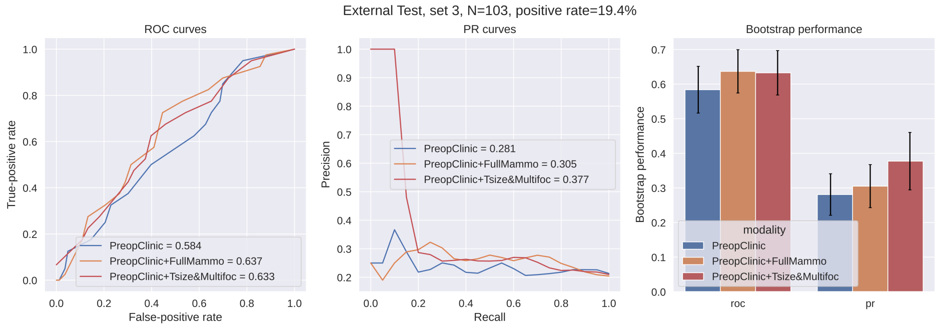

Supplementary Figure 2: Model performance of LNM prediction in the external test set. Mammograms enhanced preoperative prediction of LNM in the external test set despite the clinical variables were less representative with low predictive ability. Comparisons of ROC curves (left) , PR curves (middle) and ROC AUCs (right) between models using various modalities in the external test set are presented. Mean and standard deviation (error bars) were calculated across 1,000 bootstrap samples. LNM, lymph node metastasis; N, number of patients, PreopClinic, preoperative clinicopathology; fullMammo, full-breast mammogram; Tsize, tumor size; Multifoc, multifocality; ROC, receiver operating characteristic; PR, precision-recall; AUC, area under the curve.

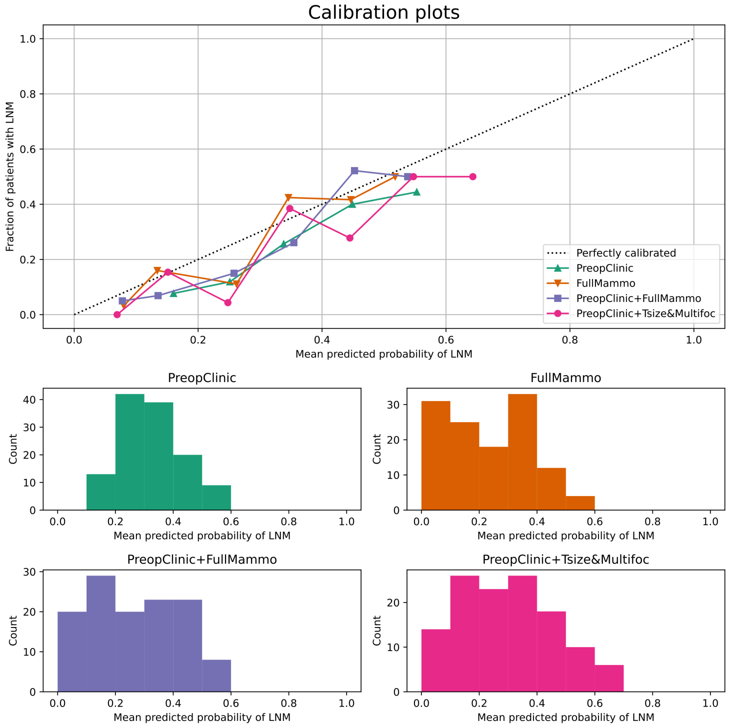

Supplementary Figure 3: Calibration curves and histograms of the distributions of the predicted probabilities. Comparisons were made across four models using different modalities in the independent test set. All models focused on calibration for low risk, i.e. the lower left corner. Prediction larger than 0.7 is missing for all models due to imbalanced classification with prevalence of positive nodes at 22%. Compared to models utilizing mammogram features (FullMammo and PreopClinic+FullMammo), models using only clinicopathological data (PreopClinic and PreopClinic+Tsize&Multifoc) were less well calibrated - under-confident for negative predictions, meaning less certain in predicting probabilities lower than 0.1. The [0,1] interval was uniformly discretized into ten bins with an interval of 0.1. PreopClinic, preoperative clinicopathology; fullMammo, full-breast mammogram; Tsize, tumor size; Multifoc, multifocality; LNM, lymph node metastasis.

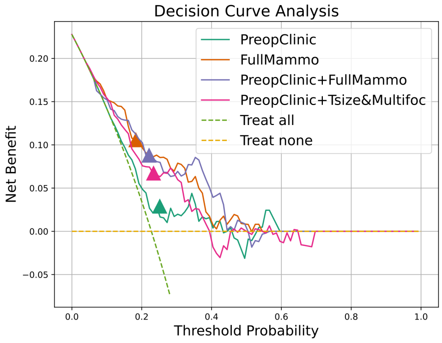

Supplementary Figure 4: Decision curves of the developed models. Comparisons were made across four models using different modalities in the independent test set. Compared to using clinicopathological data only, incorporating mammogram features significantly improved the net benefit. Triangle markers indicate the net benefit at each model's threshold where sensitivity is 91% , comparable to that of SLNB. All the four models show higher net benefit than the extreme options treat all and treat none over a wide range of clinically relevant thresholds ranging from 10% to 40%. Treat all, assume all patients have node metastasis and treat all with SLNB; Treat none, assume no patient has node metastasis and treat no with SLNB; PreopClinic, preoperative clinicopathology; fullMammo, full-breast mammogram; Tsize, tumor size; Multifoc, multifocality.

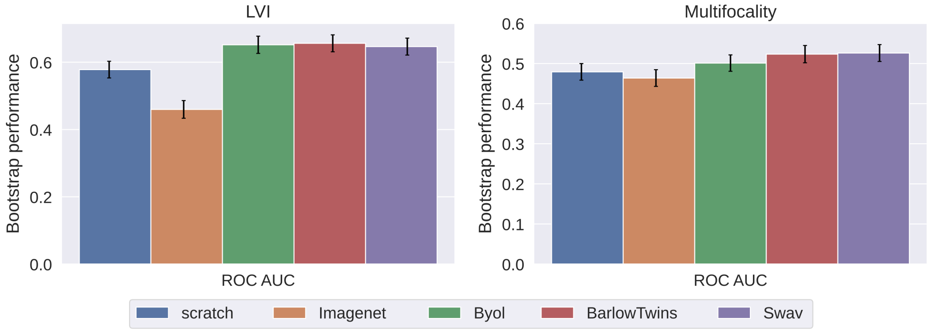

Supplementary Figure 5: Evaluation of three state-of-the-art self-supervised learning (SSL) methods in predicting LVI and multifocality. SSL on unlabeled mammograms enhanced the representations for predicting cancer outcomes. The mean and standard deviation (error bars) were calculated across 1,000 bootstrap samples. LVI, lymphovascular invasion; ROC AUC, area under the receiver operating characteristics curve.

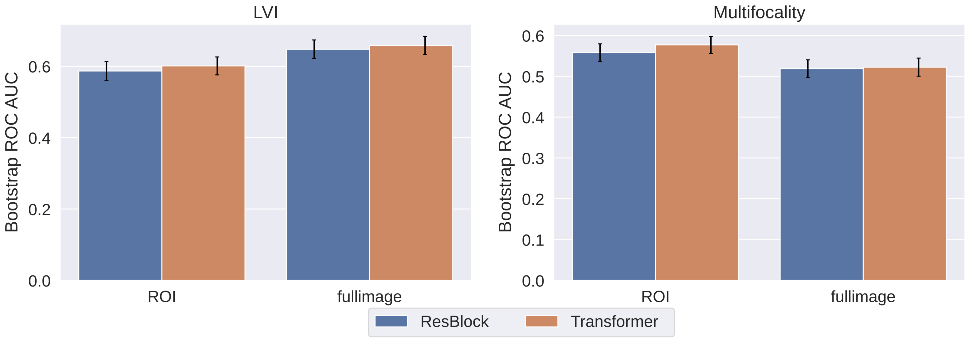

Supplementary Figure 6: Comparison of Transformer and ResBlock for mammogram modeling. Transformer outperformed ResBlock in full-breast and ROI-based mammogram modeling for LVI and multifocality prediction. The mean and standard deviation (error bars) were calculated across 1,000 bootstrap samples. ROI, region of interest; LVI, lymphovascular invasion; ROC, receiver operating characteristics; AUC, area under the curve.

Supplementary Table 3: Additional cancer-free mammograms did not improve the representation learning for cancer-related downstream tasks

SSL dataset	Linear Evaluation				
	LNM (ROC ↑)	LVI (ROC ↑)	Multifocality (ROC ↑)	Tumor size (R ↑)	Number of LNMs (R ↑)
cancer	**0.630** (±.018)	**0.639** (±.025)	**0.525** (±.021)	**0.312** (±.022)	0.116 (±.019)
cancer/ cancer-free	0.626 (±.018)	0.633 (±.025)	0.496 (±.020)	0.309 (±.023)	**0.134** (±.019)

Results suggested that such a distribution shift introduced by cancer-free patterns canceled the benefit of enhanced diversity. Another possible explanation could be that diversity introduced by cancer-free breasts of non-bilateral breast-cancer patients, which comprised the vast majority, was sufficient, so that 14k cancer-free women provided no extra benefits. BYOL was utilized for the self-supervised learning on unlabeled mammograms, both with or without an additional 14k cancer-free cases. Double cross-validation was performed for linear evaluation, where the pretrained backbone was frozen, to predict five cancer outcomes. Cancer dataset: 3,370 patients and 240,524 patches; Cancer/cancer-free dataset: 18,039 patients, 568,805 patches; LNM, lymph node metastasis; LVI, lymphovascular invasion; ROC: receiver operating characteristic; R: Pearsonr correlation coefficient.

Supplementary Table 4: Routine mammograms did not enhance overall performance and metrics predicting LVI at the sensitivity of 89%

Metrics	PreopClinic	FullMammo	PreopClinic +FullMammo	PreopClinic +Tsize&Multifoc
ROC AUC	0.763 (±0.046)	0.662 (±0.055)	0.764 (±0.050)	**0.774** (±0.045)
PR AUC	0.518 (±0.069)	0.474 (±0.069)	**0.537** (±0.075)	0.523 (±0.068)
Sensitivity (recall, TPR), %	89.3 (±2.3)	89.4 (±2.3)	89.3 (±2.3)	89.2 (±2.1)
Specificity (TNR), %	53.6 (±11.9)	28.5 (±10.6)	47.1 (±17.2)	**62.2** (±17.2)
PPV (precision), %	42.5 (±5.7)	32.0 (±3.4)	40.3 (±8.9)	**48.8** (±8.8)
NPV, %	92.7 (±3.1)	86.6 (±5.4)	91.5 (±3.2)	**93.4** (±2.9)
Accuracy, %	63.3 (±8.5)	44.9 (±7.6)	58.6 (±12.3)	**69.5** (±12.3)

Mean and standard deviation was calculated on 1,000 bootstrap samples. The best mean value was denoted in bold among all models. PreopClinic, preoperative clinicopathology; fullMammo, full-breast Mammogram; Tsize, tumor size; Multifoc, multifocality; ROC, receiver operating characteristics; AUC, area under the curve; PR, precision recall; TNR, true negetive rate; PPV, positive predictive value; NPV, negative predictive value.

Supplementary Table 5: Performance of mammogram-based Transformer in the independent test set for five cancer-related tasks.

Model	LNM (ROC ↑)	LVI (ROC ↑)	Multifocality (ROC ↑)	Tumor size (R ↑)	Number of LNMs (R ↑)
ROIMammo	0.742 (±.053)	**0.674** (±.055)	**0.517** (±.059)	0.578 (±.040)	0.219 (±.047)
FullMammo	**0.776** (±.045)	0.670 (±.054)	0.442 (±.061)	**0.620** (±.035)	**0.258** (±.040)

LNM: lymph node metastasis; LVI: lymphovascular invasion; ROIMammo: ROI-based mammogram model; FullMammo: full-breast mammogram model; ROC: receiver operating characteristic; R: Pearsonr correlation coefficient.

Supplementary References

[1] Grill, J.-B. *et al.* Bootstrap your own latent-a new approach to self-supervised learning. *Advances in neural information processing systems* **33**, 21271–21284 (2020).

[2] Zbontar, J., Jing, L., Misra, I., LeCun, Y. & Deny, S. *Barlow twins: Self-supervised learning via redundancy reduction.* 12310–12320 (PMLR, 2021).

[3] Caron, M. *et al.* Unsupervised learning of visual features by contrasting cluster assignments. *Advances in neural information processing systems* **33**, 9912–9924 (2020).

[4] Raghu, M., Zhang, C., Kleinberg, J. & Bengio, S. Transfusion: Understanding transfer learning for medical imaging. *Advances in neural information processing systems* **32** (2019).

[5] Contributors, M. Openmmlab's pre-training toolbox and benchmark. https://github.com/open-mmlab/mmpretrain (2023).

[6] You, Y., Gitman, I. & Ginsburg, B. Large batch training of convolutional networks. *arXiv preprint arXiv:1708.03888* (2017).

[7] Loshchilov, I., Hutter, F. *et al.* Fixing weight decay regularization in adam. *arXiv preprint arXiv:1711.05101* **5** (2017).

[8] Miller, J. D. *et al.* Self-supervised deep learning to enhance breast cancer detection on screening mammography. *arXiv preprint arXiv:2203.08812* (2022).
